# Cortical GABAergic Neuron Dysregulation in Schizophrenia Is Age Dependent

**DOI:** 10.1016/j.bpsgos.2025.100606

**Published:** 2025-09-08

**Authors:** Daniel Kiss, Xiaolin Zhou, Nicole Endresz, Keon Arbabi, Alex Gonzalez Segura, Daniel Felsky, Andreea O. Diaconescu, Etienne Sibille, Shreejoy J. Tripathy

**Affiliations:** a Krembil Centre for Neuroinformatics, Centre for Addiction and Mental Health, Toronto, Ontario, Canada; b Campbell Family Mental Health Research Institute, Centre for Addiction and Mental Health, Toronto, Ontario, Canada; c Institute of Medical Sciences, Temerty Faculty of Medicine, University of Toronto, Toronto, Ontario, Canada; d Department of Physiology, University of Toronto, Toronto, Ontario, Canada; e Bipolar and Depressive Disorders Unit, Hospital Clínic de Barcelona, Barcelona, Spain; f Department of Psychiatry, University of Toronto, Toronto, Ontario, Canada; g Division of Biostatistics, Dalla Lana School of Public Health, University of Toronto, Toronto, Ontario, Canada; h Department of Anthropology, University of Toronto, Toronto, Ontario, Canada; i Department of Pharmacology and Toxicology, University of Toronto, Toronto, Ontario, Canada

**Keywords:** Aging, Computational neurogenomics, Interneurons, RNA-seq, Schizophrenia, Single-cell RNA-seq

## Abstract

**Background:**

Cortical GABAergic (gamma-aminobutyric acidergic) neuron dysregulation is implicated in schizophrenia (SCZ), but it remains unclear whether these changes are due to altered cell proportions or per-cell changes in messenger RNA (mRNA) expression.

**Methods:**

We analyzed 14 bulk and cell type–specific RNA sequencing (RNA-seq) datasets from 1408 individuals (672 SCZ cases, 736 controls) across 3 neocortical regions. We deconvolved GABAergic cell-subtype proportions from bulk RNA-seq and benchmarked them against single-nucleus RNA-seq and stereological densities from matched donors. We assessed SCZ- and age-associated changes in cell proportions and per-cell gene expression.

**Results:**

SCZ was associated with altered proportions of neocortical parvalbumin (PVALB) and somatostatin (SST) cells, depending on the subject’s age at death. Younger SCZ cases (age < 70 years) showed reduced PVALB and SST cell proportions, while older cases showed unchanged or increased proportions compared with controls. Earlier-onset SCZ, associated with more severe clinical symptoms, was linked to greater reductions in these cell types. Additionally, there was robust evidence for reduced per-cell SST and vasoactive intestinal peptide mRNA among younger cases with SCZ.

**Conclusions:**

These findings suggest that SCZ is associated with complex, age-dependent alterations in GABAergic neurons, particularly affecting PVALB and SST cells. Our study underscores the importance of age-stratified analyses in SCZ, suggesting that distinct pathological processes underlie GABAergic neuron dysregulation across different age and symptom-severity groups and warranting tailored therapeutic approaches.

## Plain Language Summary

Scientists disagree about whether the brain’s inhibitory cells change in schizophrenia (SCZ). We analyzed data from over 1400 donated human brain samples, using bulk and single-cell RNA sequencing and cell imaging. We found that changes in SCZ depend strongly on participant age: People who died before 70 and had earlier-onset illness showed clear losses and lower activity of inhibitory cells, whereas those who died after 70 showed little or no loss. These results explain previous conflicting reports and highlight that age must be considered when studying, diagnosing, and treating schizophrenia.

Schizophrenia (SCZ) is a debilitating neuropsychiatric condition affecting approximately 1% of North Americans (1). SCZ is characterized by positive symptoms (e.g., delusions, hallucinations), negative symptoms (e.g., avolition, asociality), and cognitive impairments (1, 2, 3, 4). The SCZ phenotype is heterogeneous, with factors such as age of onset, sex, genetics, and lifestyle impacting severity and prognosis (5,6). On average, in North America, SCZ is associated with a decreased life expectancy, with an earlier diagnosis leading to more severe symptoms and cognitive impairments (7, 8, 9, 10).

Studies have explored the associations between SCZ and cell type–specific abnormalities in the brain, particularly in cortical GABAergic (gamma-aminobutyric acidergic) signaling networks (11, 12, 13). Changes in the functioning of GABAergic neurons have been linked to working memory and cognitive impairments in SCZ (11), contributing to a wide range of symptoms through imbalances in excitation and inhibition. Alterations in 2 particular GABAergic neuron subtypes—parvalbumin (PVALB)- and somatostatin (SST)-expressing neurons—are most strongly associated with SCZ (11,14,15). Fast-spiking PVALB neurons regulate the activity of pyramidal neurons by providing inhibition and controlling the precise timing of neural firing within cortical circuits (16,17). They are implicated in the maintenance of gamma oscillations in the dorsolateral prefrontal cortex (DLPFC) and are closely tied to cognitive processes, including working memory and cognitive flexibility (18). The DLPFC and cingulate cortex have been specifically implicated in SCZ pathology, with functional magnetic resonance imaging studies showing abnormal activations in these areas thought to underlie cognitive impairments in SCZ (19). SST neurons provide dendritic inhibition to pyramidal neurons and modulate theta network oscillations (18,20). Deficits in SST neurons in mammals have been linked to dysregulated attention, episodic memory, and spatial navigation (11,14,20,21) and have been observed across multiple brain disorders as well as in healthy aging (22, 23, 24), suggesting SST cell-intrinsic vulnerability (25).

Despite evidence implicating GABAergic neuron alterations in SCZ, it remains unclear whether these associations are the result of cell type–specific molecular (e.g., messenger RNA [mRNA]) alterations or changes in cellular abundance and density. For example, a meta-analysis by Kaar *et al.* (26) supports reduced PVALB cell density in SCZ and suggests that PVALB mRNA levels within PVALB cells are not significantly changed. Similarly, Toker *et al.* (27) found decreases in PVALB cell proportions in SCZ through a large transcriptomic meta-analysis primarily using bulk tissue deconvolution. More recently, Batiuk *et al.* (28) utilized single-nucleus RNA sequencing (snRNA-seq) to reveal a decreased abundance of specific GABAergic neuron subtypes in SCZ, particularly upper-layer SST and PVALB cells. In contrast to observations of altered cell-type densities in SCZ, a recent study by Dienel *at al.* (29) used in situ hybridization techniques to quantify the density of PVALB- and SST-expressing cells as well as the endogenous mRNA expression of PVALB and SST mRNA levels in these cells. Altered neuron cell densities were not observed, whereas PVALB and SST mRNA per cell were found to be reduced in SCZ. These findings are consistent with other studies that have found evidence for reduced PVALB mRNA expression per cell but unchanged cellular densities in SCZ (30, 31, 32).

Sabunciyan (33) identified interactions between age and diagnosis in SCZ at the transcriptomic level, showing that gene expression differences between SCZ cases and controls vary with age. Specifically, this study suggests that the most considerable SCZ-associated differential expression (DE) occurs in midlife (ages 40–60 years), diminishes in late life (ages >60 years), and may contribute to premature brain aging in SCZ (34,35). Similarly, recent studies, including our own, have linked healthy aging to reductions in SST and PVALB neurons (22). Given the parallels between SCZ and aging, particularly in cell-type proportion (CTP) changes, it is crucial to investigate age-disease interactions in SCZ. Notably, the association between SCZ-related PV and SST neuron pathology and factors such as age of onset, age at death, and disease severity remains unexplored.

In this study, we aimed to clarify the contributions of cell type–specific molecular alterations and changes in cellular proportions to the pathophysiology of SCZ, with a particular focus on PVALB and SST neurons. We hypothesized that the dysregulation of these neurons in SCZ is age dependent, with distinct patterns of cell proportion and mRNA expression changes across different age groups. Additionally, we explored the relationship between these alterations and clinical factors such as age of SCZ onset and age at death, positing that early-onset SCZ may be associated with more pronounced GABAergic neuron deficits. By integrating data from bulk and cell type–specific RNA-seq, we seek to provide a more nuanced understanding of the molecular and cellular mechanisms underlying SCZ and how they interact with the aging process.

## Methods and Materials

### Data Sources

We assembled 14 adult postmortem transcriptomic datasets that together span 8 institutional cohorts or brain banks collected as part of the PsychENCODE and CommonMind consortia (University of Pittsburgh [Pitt], Mount Sinai School of Medicine [MSSM], University of Pennsylvania [Penn], National Institute of Mental Health [NIMH] Human Brain Collection Core [HBCC]/Lieber Institute for Brain Development [LIBD], Stanley Medical Research Institute [SMRI], McLean Hospital) (36, 37, 38, 39, 40, 41) and the open-access Batiuk and Fröhlich studies (28,36). All donors were classified as either SCZ or neurotypical control and were 15 to 90+ years old at death; samples from individuals diagnosed with other conditions were excluded. These datasets cover 3 neocortical regions—DLPFC, anterior cingulate cortex (ACC), and orbital frontal cortex (OFC). These datasets comprise 3 assay types: bulk RNA-seq, snRNA-seq, and fluorescence in situ hybridization followed by laser-capture microdissection RNA-seq (FISH + LCM-seq).

Table 1 summarizes the key metadata, and Figure S1 depicts interdataset donor overlap (e.g., bulk RNA-seq and snRNA-seq collected from the same donor as part of different studies). Because the 4 SMRI subcohorts in BrainGVEX were modest in size, we combined them into a single cohort (GVEX). A subset of Pitt donors contributed both ACC bulk RNA-seq and subgenual ACC (sgACC) FISH + LCM-seq data. Tissue preparation and RNA-seq protocols differed between studies and cohorts, and details on these have been published previously (28,37, 38, 39, 40, 41, 42, 43). Distributions of key covariates, including age at death, postmortem interval (PMI), and gender, can be found in Figure S2, which summarizes metadata across all cohorts to aid in cross-dataset comparisons.

**Table 1:** Summary of RNA Sequencing Datasets

Dataset/Cohort Name	Brain Area	Cohort/Brain Bank	Male	Age, Years, Mean (SD)	RNA Type	Cases, *n*	Controls, *n*	Synapse ID
MSSM, Bulk	DLPFC	MSSM	59.6%	72.8 (14.6)	Bulk	149	163	syn22344686
Penn	DLPFC	Penn	43.2%	74.2 (13.9)	Bulk	58	37	syn22344686
Pitt	DLPFC	Pitt	79.2%	48.7 (13.9)	Bulk	57	49	syn22344686
NIMH_HBCC	DLPFC	HBCC/LIBD	71.3%	43.9 (16.6)	Bulk	96	186	syn22344107
LIBD_szControl	DLPFC	HBCC/LIBD	67.1%	44.9 (16.2)	Bulk	175	223	syn22344107
GVEX	DLPFC	SMRI	75.1%	44.5 (10.3)	Bulk	94	75	syn4590909
Pitt_ACC	ACC	Pitt	71.8%	47.9 (13.8)	Bulk	58	78	syn22344686
MSSM_ACC	ACC	MSSM	58.6%	73.1 (14.6)	Bulk	130	143	syn22344686
Penn_ACC	ACC	Penn	45.5%	73.8 (13.8)	Bulk	43	23	syn22344686
McLean	DLPFC	McLean	66.3%	63.2 (16.9)	snRNA-seq	24	24	syn25946131
MSSM, snRNA	DLPFC	MSSM	50.0%	72.7 (16.5)	snRNA-seq	41	51	syn25946131
Batiuk	DLPFC	Batiuk	52.2%	65 (10.3)	snRNA-seq	14	9	NA
Fröhlich	OFC	Fröhlich	65.2%	56 (12.7)	snRNA-seq	33	36	NA
Pitt_sgACC FISH + LCM-seq	sgACC	Pitt	50.0%	48.3 (9.8)	LCM-seq	18	19	NA

Dataset/cohort name denotes name of the dataset used in this study. Brain area denotes the neocortical area sampled in the dataset. Cohort/brain bank denotes the name of the cohort and brain bank: MSSM, Penn, Pitt, HBCC/LIBD, SMRI, and McLean. RNA type denotes type of RNA-seq data collected, either bulk tissue RNA-seq, snRNA-seq, or LCM-seq. Synapse ID denotes the respective identifier at synapse.org where data were obtained.

ACC, anterior cingulate cortex; DLPFC, dorsolateral prefrontal cortex; FISH, fluorescence in situ hybridization; HBCC, Human Brain Collection Core; LCM-seq, laser-capture microdissection RNA sequencing; LIBD, Lieber Institute for Brain Development; McLean, McLean Hospital; MSSM, Mount Sinai School of Medicine; NA, not available; NIMH, National Institute of Mental Health; OFC, orbitofrontal cortex; Penn, University of Pennsylvania; Pitt, University of Pittsburgh; sgACC, subgenual ACC; SMRI, Stanley Medical Research Institute; snRNA-seq, single-nucleus RNA sequencing.

### Data Preprocessing

#### Reference Cell-Type Taxonomy

We defined cell-type identities using a reference taxonomy provided within the Allen Institute for Brain Science Multiple Cortical Areas Smart-seq snRNA-seq dataset from the Allen Brain Map portal (https://portal.brain-map.org/atlases-and-data/rnaseq/human-multiple-cortical-areas-smart-seq). Details of tissue procurement, library preparation, and clustering are provided in (44,45). The resource comprises 49,495 single nuclei collected at ultra-high sequencing depth from 6 neocortical regions—the medial temporal gyrus, ACC, and primary visual, motor, somatosensory, and auditory cortices. For the current study, we adopted the authors’ subclass annotation (field subclass_label), which offers an intermediate cell-type resolution: It divides the broad excitatory and inhibitory classes into recognizable GABAergic and glutamatergic subclasses while avoiding oversplitting. This taxonomy yields 19 distinct neocortical cell types and captures all major neuronal and non-neuronal populations expected in the human cortex.

#### Bulk RNA-Seq

Bulk RNA-seq counts for each sample were downloaded from publicly available sources and aggregated into count matrices. Matrices were converted into counts per million using the cpm() function from edgeR (46) and log_2_ transformed with a prior count of 0.1. Genes with a low standard deviation (SD < 0.1) were filtered from subsequent analyses. No additional outliers were identified at the gene expression level, and thus no samples were excluded.

#### Single-Nucleus RNA-Seq

For McLean and MSSM snRNA-seq data (41), we retrieved the processed count matrices for these from Synapse (syn25946131). Two additional snRNA-seq datasets were included in the analysis: one from Batiuk *et al.* (28), obtained from Zenodo (https://zenodo.org/record/6921620), and another from Fröhlich *et al.* (36), retrieved from the Gene Expression Omnibus (Accession No. GSE254569). For all single-nucleus datasets, only samples from donors age 15 or older were retained. Additionally, only samples diagnosed as control or SCZ were included in the analysis. For snRNA-seq data, only samples with at least 500 nuclei detected were retained. Gene-level filtering was performed to exclude low-variance genes, specifically genes with an SD <0.1 in log-transformed counts per million values were removed from the analysis. Sample-level quality control (QC) was also implemented.

Due to differences in cell-type taxonomies between snRNA-seq datasets, we mapped the cell-type definitions of each nucleus to that of our reference cell-type taxonomy described above. Reference-based label transfer was performed using Seurat (47) to project reference data onto the query object. Anchors were identified between the datasets, and the reference principal component analysis structure was projected onto the query. The TransferData() function was then used to classify query cells based on reference cell-type labels, generating predicted cell identities and scores, which were added to the query data. Donors were discarded from downstream analyses if <500 total QC-passing nuclei were sampled.

Following cell-type label transfer, we utilized the Seurat (version 5) (48) function, *AggregateExpression*, to generate robust donor-level gene expression profiles for each cell type. This approach aggregates gene expression by grouping cells according to their assigned cell-type labels. A benefit to pseudobulking is that it helps mitigate the sparsity and noise inherent in single-cell data, providing a more robust representation of gene expression at the cell-type level (49).

#### FISH + LCM-Seq

sgACC FISH-based microscopy cell densities and LCM-seq–based per-cell expression values were reanalyzed from Arbabi *et al.* (39) from donors from the Pitt cohort. FISH microscopy provided independent cell densities (FISH-labeled cells/mm^2^). Samples with cell densities >3 times the interquartile range were excluded to eliminate outliers.

### CTP Estimation and Validation

#### Bulk RNA-Seq Cell-Type Deconvolution

For bulk RNA-seq datasets, we performed relative CTP (rCTP) estimation using the mgpEstimate() function from the MarkerGeneProfile (MGP) package in R as described previously (27,48). We made use of our previous list of cell type–specific marker genes for each cell type as described previously (50); marker genes for each of the major GABAergic neuron types are listed in Table S1. MGP estimates are interpreted as rCTP estimates, which serve as a proxy for cell-type abundance in each sample. rCTPs were *z*-score normalized within each dataset for all subsequent analyses.

To facilitate comparisons with an additional deconvolution algorithm, we also estimated CTPs with the well-established dtangle algorithm (51,52). Marker gene sets were derived using the top 10% most-expressed genes per subclass using the Allen Institute for Brain Science snRNA-seq reference taxonomy (44,45) using find_markers(“ratio”). Unlike MGP, dtangle outputs absolute cell-type fractions scaled between 0 and 1.

#### Single-Nucleus CTPs

We derived single-nucleus CTPs (snCTPs) following our previously described workflow (22,50). For each donor, the nuclei assigned to a given cell type were divided by the donor’s total nuclei count, yielding absolute proportions for every cell type.

#### Cross-Modal Benchmarking of Bulk Deconvolution Methods

To evaluate the accuracy of our bulk RNA-seq deconvolution algorithms, we focused on donors for whom bulk RNA-seq and at least one additional modality were available from the same cortical region (detailed in Figure S1). Two such overlaps were identified.

1. MSSM/Ruzicka overlap: A subset of donors from the MSSM brain bank contributed bulk DLPFC RNA-seq, while the Ruzicka study (41) generated snRNA-seq from the same individuals’ PFC (Brodmann areas 9, 10, and 46).
2. Pitt overlap: A subset of donors from the Pitt brain bank had bulk ACC RNA-seq and, separately, FISH-based cell densities from sgACC collected for our previous study [Arbabi *et al.* (40)]. For this comparative benchmarking analysis, we also made use of data from an additional 19 donors with diagnosed bipolar disorder.

### Statistical Analyses

#### Association Testing for CTPs With SCZ Status

To estimate associations of SCZ case/control status and other covariates with bulk and single nucleus–based CTP values, we used several statistical models.

The base model for each analysis is as follows:

(1) CTP∼diagnosis+RIN+PMI+ageatdeath+reportedgender+dataset

where CTP denotes either relative bulk estimates (rCTPs) or absolute single-nucleus proportions (snCTPs). RNA integrity number (RIN) was omitted for snRNA-seq datasets in which it was unavailable. Continuous covariates (e.g., PMI) were *z* standardized prior to analyses.

#### Bulk rCTP Analyses

For bulk rCTPs, we first fitted a separate linear model for every dataset and cell type in both the DLPFC and ACC. Next, we conducted 2 pooled mega-analyses: one that binned donors by decade of death (15–29 years up to 90+ years) and a second that compared 2 broader age groups, younger than 70 years versus 70 years and older. To test whether the SCZ effect varied with age, we added an age × diagnosis interaction term to the pooled model. Finally, covariate-residualized rCTPs were contrasted between cases and controls with 2-sided Mann-Whitney *U* tests (53).

#### snCTP Analyses

Because sample sizes were generally much smaller among snRNA-seq datasets, snCTPs were analyzed only in the <70 years and ≥70 years age at death strata using the same models above but excluding RIN.

#### FISH-Based Microscopy Analyses

sgACC cell densities (cells/mm^2^) from Arbabi *et al.* (40) were compared between donors with SCZ and control donors with 2-sided Wilcoxon rank-sum tests. Age stratification between the broad age group strata above was not possible as the oldest donor in this dataset was 68 years at death.

#### SCZ Age of Onset Analyses

In our analyses, 3 datasets, GVEX, LIBD_szControl, and the Batiuk dataset, had available information on the age of SCZ onset for SCZ cases. For these analyses, for the GVEX and LIBD_szControl datasets, rCTPs were first residualized for RIN, PMI, age at death, and gender prior to association with SCZ age of onset using Pearson’s *r*. For the Batiuk dataset, the snCTPs were directly associated with SCZ age of onset.

#### Cell Type–Specific DE

Cell type–specific SCZ-related DE was quantified for snRNA-seq using pseudobulked gene expression profiles per donor and cell type. We excluded data from donors with fewer than 1000 total QC-passing nuclei per sample. DE was tested for separately among the broad age strata defined above. Limma-voom and edgeR (54,55) were used for DE testing using the following model:

(2) $$\text{gene}\;\text{expression}\;∼\;\text{diagnosis}+\text{PMI}+\text{age}\;\text{at}\;\text{death}+\text{reported}\;\text{gender}+\log_{10}\left(\text{nuclei per}\;\text{donor}\right)$$

SCZ diagnosis was the main predictor, while PMI, age, gender, cohort, and total counts of nuclei sampled per donor were included as covariates. Summary statistics of cell type–specific DE from our sgACC FISH + LCM-seq dataset (40) were incorporated unchanged.

To meta-analyze cell type–specific DE results across datasets, dataset-specific log_2_ fold changes (FCs) and standard errors were combined via a random-effects meta-analysis using the rma function in the R package metafor (56), using restricted maximum likelihood estimation, producing weighted, heterogeneity-adjusted estimates of cell type–specific transcriptional differences associated with SCZ.

#### Multiple Comparisons Consideration

For each model, adjusted *p* values were obtained using false discovery rate (FDR) correction (57) to account for comparisons across multiple cell types and multiple genes.

## Results

### Proportions of GABAergic Neuron Subtypes Can Be Deconvolved From Human Brain Bulk RNA-Seq Data

Given the abundance of brain bulk RNA-seq datasets from postmortem SCZ cases and controls, we first assessed the accuracy of cell-type deconvolution methods for estimating GABAergic neuron subtype proportions. We compared deconvolution results using the MGP (27,48) and dtangle (51) methods with gold-standard CTPs from snRNA-seq and microscopy-based cell densities.

**Figure 1:**
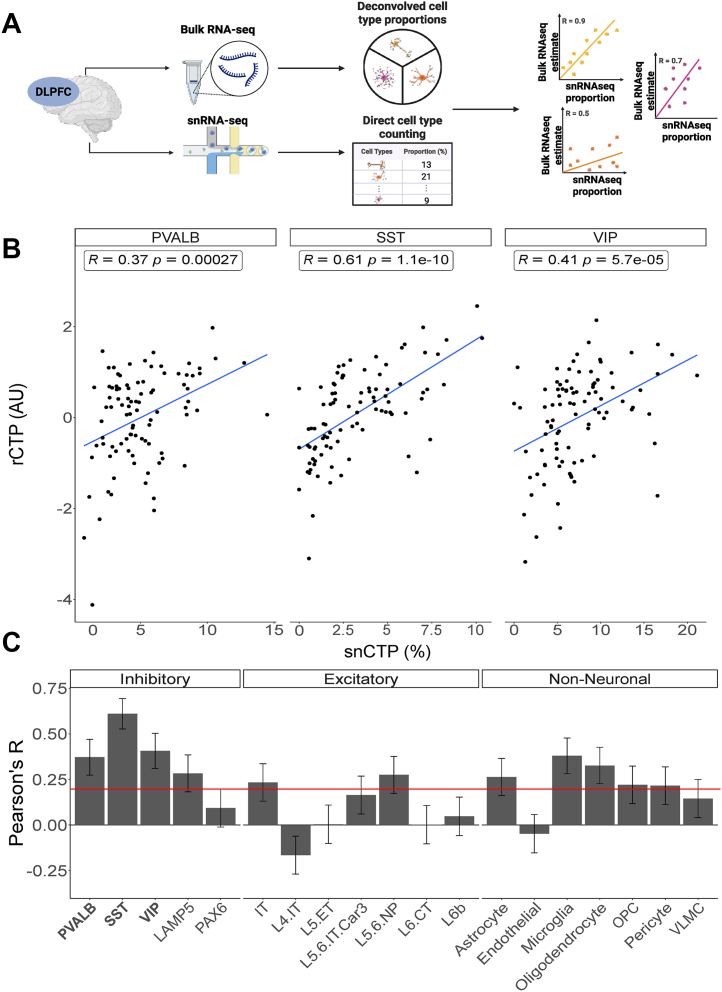
Concordance between cell proportion estimates from bulk tissue deconvolution and snRNA-seq from matched tissue samples. **(A)** Schematic representation of bulk RNA-seq collection and cell proportion estimation via deconvolution (top), alongside snRNA-seq with direct cell proportion calculation through cell counting (bottom) for DLPFC samples from matched donors. The rightmost panel shows the calculation of concordance between bulk deconvolution and snRNA-seq proportions using correlation coefficients. Each dot represents a distinct individual, and colors indicate different cell types. **(B)** Scatterplots showing the correlation between snCTPs (x-axis, % of total cells in the sample) and rCTPs (y-axis, AUs) derived from bulk tissue deconvolution. Inset values show Pearson’s correlation coefficient (*r*) and corresponding *p* values. **(C)** Bar plot displaying Pearson’s *r* values for various cell types (x-axis). Error bars represent the SEM. AU, arbitrary unit; DLPFC, dorsolateral prefrontal cortex; OPC, oligodendrocyte precursor cell; PVALB, parvalbumin; rCTP, relative cell-type proportion; snCTP, single-nucleus CTP; snRNA-seq, single-nucleus RNA sequencing; SST, somatostatin; VIP, vasoactive intestinal peptide; VLMC, vascular leptomeningeal cells.

Using data from the MSSM cohort that included both bulk RNA-seq and snRNA-seq collected from the DLPFC of the same donors (41), we observed that MGP-based rCTPs were correlated with snCTPs for PVALB, SST, and vasoactive intestinal peptide (VIP) neurons, with Pearson’s *r* values of 0.37, 0.61, and 0.41, respectively (Figure 1B). Moreover, across all cell types, we found that MGP-based rCTPs were usually positively correlated with snCTPs (Figure 1C). We found qualitatively similar findings using dtangle, an alternative method for cell-type deconvolution (Figure S3A), and in modified versions of our marker gene lists that excluded the eponymous marker genes (e.g., PVALB mRNA for PVALB cells) (Figure S3B, C) (34,50). Correlation analyses comparing bulk-derived estimates with matched snRNA-seq data showed that the MGP-inferred abundances for PVALB, SST, and VIP neurons were largely specific to their intended subtypes (Figure S4). On the basis of these observations, together with our previous usage and benchmarking of the MGP algorithm in Alzheimer’s disease and normative aging cohorts (34,48), we adopted MGP as our primary deconvolution tool for the current work.

We further validated bulk tissue deconvolution against microscopy-based cell densities using datasets from the Pitt cohort (40), where we previously used stereological methods to quantify PVALB, SST, and VIP cell densities in the subgenual ACC via FISH. Comparing these with rCTPs derived from bulk RNA-seq data from the same donors, we found Pearson’s *r* values of 0.34, 0.40, and 0.38 for PVALB, SST, and VIP, respectively (Figure S5), providing modest corroboration of our bulk deconvolution estimates against these gold-standard cell counts (Figure S5).

In summary, while acknowledging the inherent limitations of bulk deconvolution (see Discussion), our rigorous multimethod and multicohort validation suggests that bulk neocortex RNA-seq–based deconvolution provides useful proxies for GABAergic cell-subtype proportions, allowing for the assessment of interindividual differences in these cell populations and their association with neuropsychiatric disease.

### Cohort-Dependent Differences in PVALB and SST Cell Proportions in SCZ

To assess whether SCZ is associated with differences in GABAergic neuron subtype proportions, we analyzed 9 bulk RNA-seq datasets from 5 postmortem cohorts within the CommonMind and PsychENCODE consortia (Table 1). Bulk RNA-seq data were collected from the DLPFC or ACC. We used the MGP algorithm to estimate CTPs for each major cell type in each dataset.

**Figure 2:**
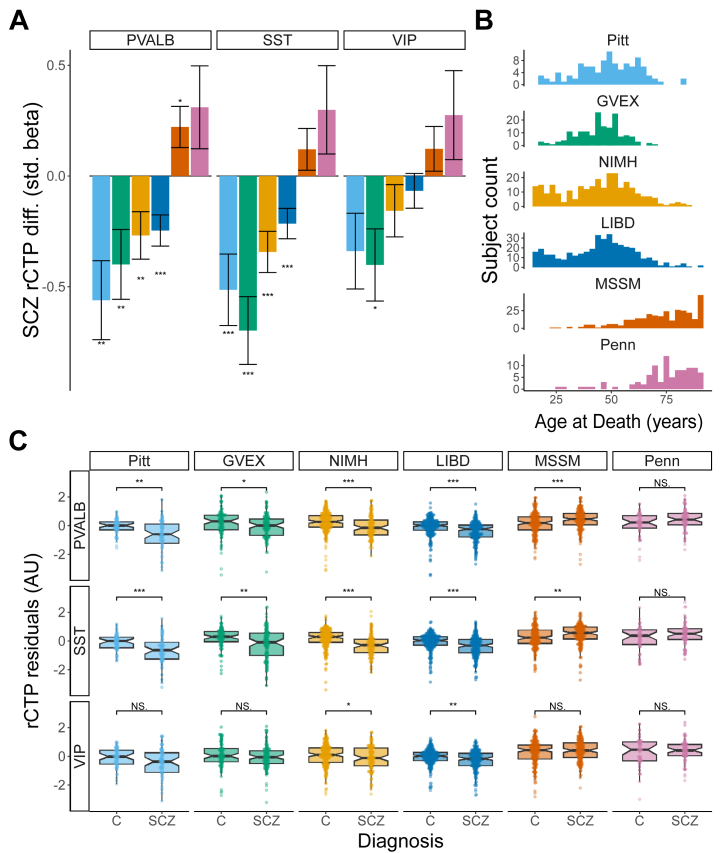
Differences in DLPFC GABAergic neuron proportions between SCZ cases and controls. **(A)** Bar plots showing standardized beta coefficients (βs) for differences in rCTPs of PVALB, SST, and VIP neurons between SCZ and control groups across 6 bulk RNA sequencing datasets. Positive values indicate increased proportions in SCZ, while negative values indicate decreases. Error bars represent standard errors, and asterisks indicate significance based on false discovery rate correction (∗*p*_FDR_ = .1, ∗∗*p*_FDR_ = .05, ∗∗∗*p*_FDR_ = .01). **(B)** Age at death distribution for each dataset. Datasets are ordered by increasing mean age in years. **(C)** Box plots of residualized rCTPs, after controlling for demographic and technical covariates, for PVALB, SST, and VIP neurons across datasets. AU, arbitrary unit; C, control; DLPFC, dorsolateral prefrontal cortex; GABAergic, gamma-aminobutyric acidergic; LIBD, Lieber Institute for Brain Development; MSSM, Mount Sinai School of Medicine; NIMH, National Institute of Mental Health; NS, nonsignificant; Penn, University of Pennsylvania; Pitt, University of Pittsburgh; PVALB, parvalbumin; rCTP, relative cell-type proportion; SCZ, schizophrenia; SST, somatostatin; VIP, vasoactive intestinal peptide.

First, we examined how rCTPs differed between SCZ cases and controls, adjusting for demographic and technical factors (Figure 2A). SCZ was associated with reduced PVALB and SST rCTPs in 4 of the 6 DLPFC datasets (GVEX, NIMH, Pitt, and LIBD) at FDR < 0.10. In the Penn cohort, PVALB and SST rCTPs did not differ significantly between SCZ cases and controls, while the MSSM cohort showed increased PVALB rCTPs in SCZ cases but no change in SST rCTPs. VIP cells generally showed no significant differences, except for decreased rCTPs in the NIMH dataset (β = −0.40, FDR = 0.053). Similar patterns were observed in the ACC datasets, with SCZ-associated reductions in PVALB, SST, and VIP cells in the Pitt ACC dataset and no significant changes in the MSSM or Penn ACC datasets (Figure S6).

When we investigated factors that might explain these differing results, we found that the cohorts varied widely in average subject age at death (Figure 2B). Datasets with younger donors (GVEX [mean age at death = 44.5], NIMH [mean age at death = 43.9], Pitt [mean age at death = 48.7], and LIBD [mean age at death = 44.9]) tended to show SCZ-associated reductions in PVALB and SST rCTPs, whereas those with older donors (MSSM [mean age at death = 72.8] and Penn [mean age at death = 74.2]) showed unchanged or increased rCTPs (Figure S7). These results suggest that age is a significant factor in whether PVALB and SST rCTPs are observed as increased or decreased in SCZ.

### SCZ-Associated Changes in PVALB and SST Cell Proportions Vary With Subject Age

**Figure 3:**
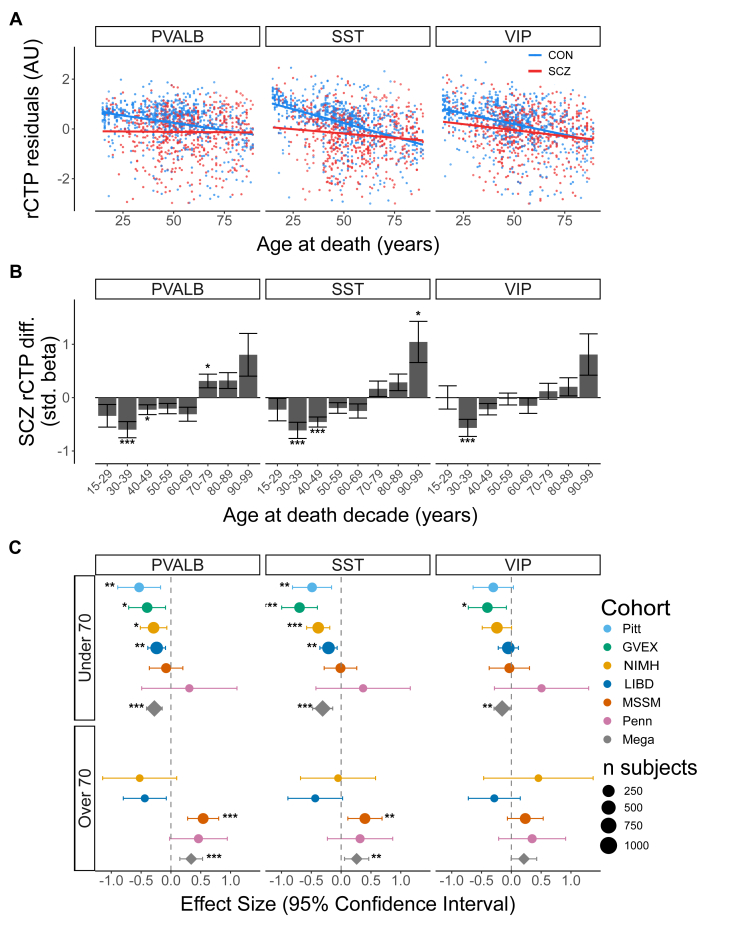
Age modulates SCZ-related differences in dorsolateral prefrontal cortex GABAergic cell proportions. **(A)** Scatterplots illustrate the association between age at death and relative proportions of PVALB, SST, and VIP-expressing neurons in SCZ cases versus controls. Each point reflects a single bulk RNA sequencing sample and has been residualized for cohort and demographic and technical differences; solid lines show the best-fit linear regression for each diagnostic group. **(B)** Decade-binned bar plots display the standardized β coefficients for the effect of SCZ per decade after accounting for technical and biological covariates. Error bars represent standard errors, and asterisks indicate significance based on false discovery rate correction (∗*p*_FDR_ = .1, ∗∗*p*_FDR_ = .05, ∗∗∗*p*_FDR_ = .01). **(C)** Forest plot summarizing the same SCZ effect after stratifying donors into under 70 and over 70 age strata. Diamonds represent the pooled (mega-analysis) effect estimate; circles represent cohort-specific estimates. Horizontal bars are 95% CIs, and point size is proportional to cohort sample size. Asterisks positioned to the left (negative effect) or right (positive effect) of each bar follow the same false discovery rate thresholds as in panel **(B)**. AU, arbitrary unit; CON, control; GABAergic, gamma-aminobutyric acidergic; LIBD, Lieber Institute for Brain Development; MSSM, Mount Sinai School of Medicine; NIMH, National Institute of Mental Health; Penn, University of Pennsylvania; Pitt, University of Pittsburgh; PVALB, parvalbumin; rCTP, relative cell-type proportion; SCZ, schizophrenia; SST, somatostatin; VIP, vasoactive intestinal peptide.

Given our observation that SCZ-related differences in GABAergic cell rCTPs may be age dependent, next we examined this relationship more systematically across the adult lifespan. We combined rCTPs across each bulk RNA-seq DLPFC (Figure 3A) and ACC dataset (Figure S9), adjusting for technical covariates. Consistent with earlier reports from us and others (22,23), control samples displayed a gradual, almost linear cross-sectional decline in PVALB, SST, and VIP proportions with advancing age at death. In contrast, rCTPs from SCZ cases started from a markedly lower baseline than controls in early adulthood, but their downward slope with advancing age was considerably more shallow, gradually converging with rCTPs from control samples in the later decades of life.

To more carefully quantify these age-related differences in GABAergic rCTPs between SCZ cases and controls, we used 2 approaches. First, using decade-binned analysis (Figure 3B), we observed that SCZ was associated with reduced PVALB, SST, and VIP rCTPs in younger donors, particularly those ages 30 to 49 years at death, effects that attenuated in donors in middle age (50–69 years) at death. However, we noted that this effect reversed for the oldest donors, with associations indicating increased PVALB and SST rCTPs in the oldest donors with SCZ compared with control donors (≥70 years). We saw similar results in bulk RNA-seq data from both the DLPFC (Figure 3) and ACC (Figure S9), with PVALB and SST rCTPs being negatively associated with SCZ in younger age bins and attenuating in older age bins. The direction of change in the 2 brain regions was nearly identical, with the DLPFC showing higher significance and magnitudes of change, likely due to differences in sample size and power.

Dichotomizing donors into under 70 and over 70 age at death strata (Figure 3C) yielded opposing directions of effect across age groups. In the younger strata, we observed significant negative pooled effect sizes across all 3 neuron subtypes, with the strongest associations in PVALB (β = −0.25) and SST (β = −0.29) neurons (both FDR < 0.05). In contrast, the direction of effect was reversed in the older strata, where corresponding estimates for PVALB and SST neurons were significantly increased (β = 0.18 and β = 0.15, respectively). The magnitude of these effects varied among cohorts, with the GVEX and Pitt cohorts showing the largest differences in the younger group, whereas the MSSM cohort contributed most to the observation of increased PVALB and SST rCTPs in SCZ in late life. VIP mirrored the patterns described above but achieved statistical significance only in the younger stratum.

Because younger donors with SCZ were disproportionately more likely to have died by suicide (Figure S8), using the GVEX and LIBD_szControl cohorts where information on cause of death by suicide was available, we reperformed the analysis above after excluding donors who died by suicide. The hallmark reduction in PVALB and SST GABAergic proportions among donors with SCZ <70 years persisted with similar effect sizes, indicating that our principal findings were unlikely to be merely driven by confounds related to suicide as a cause of death (58,59).

Additionally, we tested for a statistical interaction between subject age at death and SCZ case/control diagnosis (see Methods and Materials). We observed statistically significant positive interactions between subject age at death and SCZ diagnosis for PVALB and SST rCTPs (Figure S10). Furthermore, we observed statistically significant, but weaker magnitude, interactions between age and SCZ status for rCTPs for other cell types, including L5/6 near projecting pyramidal cells, microglia, and oligodendrocyte precursor cells.

In summary, younger donors with SCZ showed reduced proportions of PVALB and SST cells, whereas older donors were more likely to exhibit increased proportions compared with control donors. These results underscore the critical importance of subject age in determining whether PVALB and SST cell proportions are decreased, unchanged, or even increased in SCZ.

### snRNA-Seq and FISH Stereology Corroborate Age-Dependent PVALB and SST Cell Proportion and Density Differences in SCZ

To validate the age-modulated neuron changes in SCZ inferred from bulk deconvolution, next we turned to more direct measurements of cell-type abundance. We used snRNA-seq data from 3 DLPFC datasets (McLean, MSSM, Batiuk) and snRNA-seq data from 1 OFC dataset (Fröhlich) and double-label FISH stereology from the Pitt cohort, which quantified PVALB, SST, and VIP cell densities in the sgACC.

**Figure 4:**
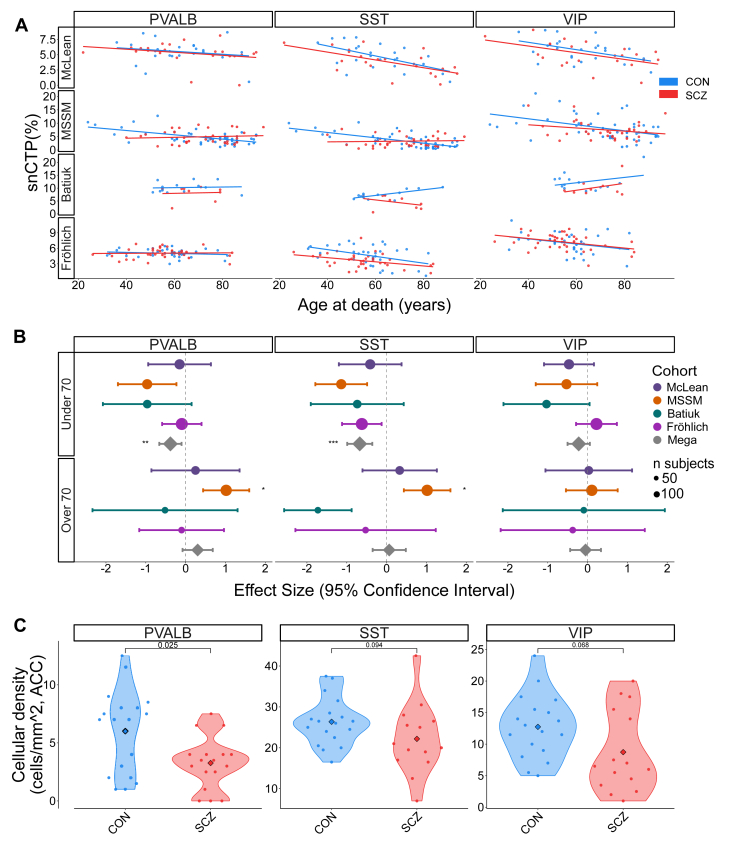
SCZ-associated differences in snCTPs of dorsolateral prefrontal cortex GABAergic neuron subtypes. **(A)** Scatterplots showing snCTPs for PVALB-, SST-, and VIP-expressing neurons in SCZ cases and controls, plotted against age at death across 4 cohorts (McLean, MSSM, Batiuk, and Fröhlich). Each point represents a donor sampled using snRNA-seq, with lines showing the best linear fit per diagnosis group. **(B)** Forest plots showing adjusted effect sizes (with 95% CIs) for the effect of SCZ on snCTPs for PVALB, SST, and VIP neurons. Results are stratified by age at death (under 70 vs. over 70 years) and displayed across 4 independent snRNA-seq datasets: McLean, MSSM, Batiuk, and Fröhlich. The bottom row (Mega) shows the pooled effect size derived from mega-analysis across all cohorts for each age group and cell type. Asterisks indicate significance based on false discovery rate correction (∗*p*_FDR_ = .1, ∗∗*p*_FDR_ = .05, ∗∗∗*p*_FDR_ = .01). The size of each point reflects the sample size of the corresponding cohort. **(C)** Violin plots show densities of PVALB, SST, and VIP neurons in SCZ cases and controls stained using double-label fluorescent in situ hybridization on subgenual ACC sections. Dots reflect per-subject quantification of counts of labeled cells expressing more than 10 or more cell type–specific marker grains per unit area. Outlier samples removed independently per group if outside range defined by 3 times the interquartile range. Inset *p* values denote Wilcoxon rank-sum tests. ACC, anterior cingulate cortex; CON, control; GABAergic, gamma-aminobutyric acidergic; McLean, McLean Hospital; MSSM, Mount Sinai School of Medicine; PVALB, parvalbumin; SCZ, schizophrenia; snCTP, single-nucleus cell-type proportion; SST, somatostatin; VIP, vasoactive intestinal peptide.

First, snRNA-seq data from control donors showed a decrease in snCTPs for older compared with younger donors (Figure 4A), consistent with our findings using bulk deconvolution. A mega-analysis that stratified donors by age at death showed that SCZ cases younger than 70 had significantly reduced PVALB and SST snCTPs relative to controls (Figure 4B). The magnitude of this difference varied among datasets: It was the largest in the Batiuk cohort, intermediate in the MSSM and McLean datasets, and smallest in the Fröhlich dataset. In the ≥70 years group, the pooled mega-analysis estimate was not significant as effect estimates were heterogeneous between cohorts, with PVALB or SST snCTPs significantly increasing in SCZ only for the MSSM cohort, consistent with our previous bulk RNA-seq–based analyses of this cohort (Figure 3C). When donors were analyzed without stratifying by age at death, no significant case-control differences emerged (Figure S11), consistent with earlier analyses performed in these single-cohort studies (41). Formal interaction tests confirmed that the impact of diagnosis lessened with age, yielding positive age × diagnosis coefficients for PVALB (β = 0.274, adjusted *p* = .033) and SST (β = 0.302, adjusted *p* = .021).

Second, using FISH-based stereology data that we had previously collected from the sgACC from donors in the Pitt cohort (Figure 4C), we confirmed reduced densities in SCZ of PVALB cells (*p* = .025) and statistically trending reductions in SST (*p* = .094) and VIP (*p* = .068) cells.

Taken together, these independent snRNA-seq and stereological measurements support the age-dependent shift that we had first detected in bulk RNA-seq. In younger adults with SCZ, PVALB and SST cell abundances were significantly reduced relative to age-matched control subjects, whereas elderly SCZ cases showed unchanged or modestly increased proportions overall relative to controls in some cohorts, such as the MSSM. A comprehensive summary of effect sizes across modalities is provided in Tables S2 to S4.

### Earlier Onset of SCZ Is Associated With Reduced PVALB and SST Cell Proportions

Next, we investigated whether clinical factors, such as the age of SCZ onset, were associated with differences in GABAergic neuron rCTPs. Although detailed clinical or symptom severity information was generally unavailable from donors in these cohorts (42), age of SCZ onset was provided in 2 bulk tissue datasets (GVEX and LIBD) and 1 single-nucleus dataset (Batiuk).

**Figure 5:**
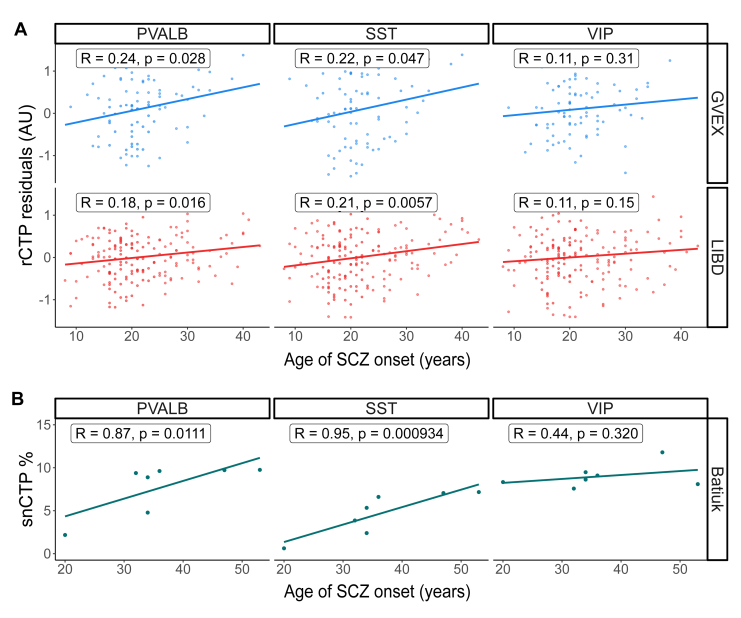
Effects of SCZ age of onset on dorsolateral prefrontal cortex neuron-subtype proportions. **(A)** Associations between age of onset of SCZ and rCTPs estimated from bulk RNA-seq data for PVALB, SST, and VIP-expressing neurons. **(B)** Associations between age of onset of SCZ and snCTPs estimated from single-nucleus RNA-seq data for the same subtypes. The y-axis in both panels shows rCTP or snCTP values after residualizing for covariates, including subject age at death. Each point represents an individual sample, with point color indicating the contributing study (Batiuk, GVEX, or LIBD). Inset lines depict the best linear fit; accompanying Pearson’s *r* and *p* values are shown. AU, arbitrary unit; LIBD, Lieber Institute for Brain Development; PVALB, parvalbumin; rCTP, relative cell-type proportion; RNA-seq, RNA sequencing; SCZ, schizophrenia; snCTP, single-nucleus CTP; SST, somatostatin; VIP, vasoactive intestinal peptide.

After accounting for age at death and other demographic and technical covariates, we found that an earlier age of onset of SCZ was associated with decreased rCTPs in PVALB and SST cells in both the GVEX and LIBD datasets (GVEX: PVALB *R* = 0.24, *p* = .028; SST *R* = 0.22, *p* = .047; LIBD: PVALB *R* = 0.18, *p* = .016; SST *R* = 0.21, *p* = .0057) (Figure 5). Similarly, an earlier age of onset was associated with decreased PVALB and SST snCTPs in the Batiuk dataset (PVALB *R* = 0.87, *p* = .0111; SST *R* = 0.95, *p* = .000934). Lastly, as SCZ age of onset is expected to be confounded with lifetime duration of SCZ, we note that we did not see significant associations between SCZ duration of illness and PVALB and SST rCTPs or snCTPs (Figure S11).

These findings show that SCZ cases with an earlier onset of SCZ, and thus potentially more severe symptomatology (1,10), are more likely to exhibit greater reductions in PVALB and SST neurons than donors with later SCZ onset. Therefore, among SCZ cases, differences in ages of SCZ onset explain some of the interindividual heterogeneity in neuron pathology.

### Cell-Intrinsic Transcriptional Changes Within GABAergic Cell Subtypes in SCZ

**Figure 6:**
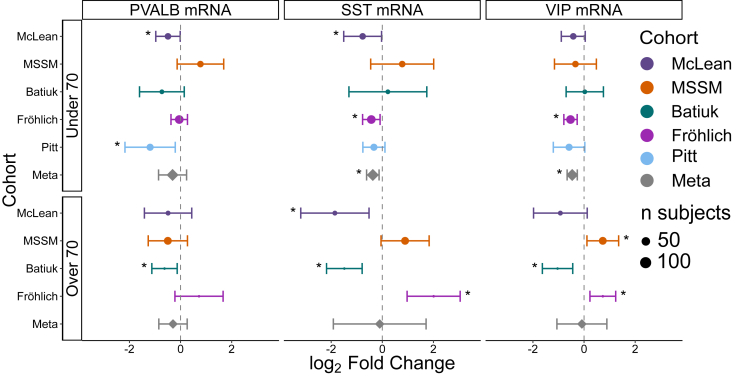
Gene expression differences in cortical GABAergic neuron marker genes in SCZ. SCZ-associated differential expression for PVALB-, SST-, and VIP-expressing mRNA in their respective cell types across 5 independent cell type–specific RNA sequencing datasets: McLean, MSSM, Batiuk, Fröhlich, and Pitt. The Meta row presents the pooled effect size from meta-analysis across all datasets for each cell type and age group. Asterisks (∗) denote significant differential expression in SCZ (unadjusted *p* < .05). Error bars represent 95% CIs. Meta-analysis values incorporate weighted effect size estimates across cohorts to account for interstudy variability. The size of each point reflects the sample size of the corresponding cohort. GABAergic, gamma-aminobutyric acidergic; McLean, McLean Hospital; mRNA, messenger RNA; MSSM, Mount Sinai School of Medicine; Pitt, University of Pittsburgh; PVALB, parvalbumin; SCZ, schizophrenia; SST, somatostatin; VIP, vasoactive intestinal peptide.

Having shown that SCZ alters the abundance of PVALB and SST cells, next we asked whether the remaining cells display parallel shifts in their own transcriptional output. To replicate the observations of Deniel *et al.* (29), we carried out cell type–specific DE meta-analyses of the canonical marker genes PVALB, SST, and VIP in these same cell types (Figure 6). We utilized single-nucleus data from the 4 frontal cortex snRNA-seq datasets (McLean, MSSM, Batiuk, and Fröhlich) with LCM-seq data from sgACC (Pitt) and stratified analyses by age at death (<70 vs. ≥70 years) to mirror our earlier proportion analyses.

In the younger stratum (<70 years), the DE meta-analysis revealed significant downregulation of SST mRNA (log_2_FC = −0.370, *p* = .0029) and VIP mRNA (log_2_FC = −0.463, *p* = 4.98 x 10^-6^) within their respective cell types, with negligible between-study heterogeneity (*I*^2^ ≈ 0%). In contrast, PVALB mRNA showed no consistent transcriptional change (log_2_FC = −0.311, *p* = .27) and displayed moderate between-study heterogeneity (*I*^2^ = 73%). In the older stratum (≥70 years), none of these mRNA markers reached significance, and all 3 exhibited substantial heterogeneity (*I*^2^ ≥ 50%), indicating that any transcriptional change in late life is cohort specific rather than universal.

Examining effects among individual datasets underscores the variability highlighted in our meta-analysis. Among younger subjects, we observed that PVALB mRNA was significantly downregulated in the McLean (log_2_FC = −0.492, *p* = .044) and Pitt (log_2_FC = −1.191, *p* = .020) datasets; SST mRNA was significantly downregulated in the McLean (log_2_FC = −0.768, *p* = .042) and Fröhlich (log_2_FC = −0.430, *p* = .015) datasets; and VIP was downregulated only in the Fröhlich dataset (log_2_FC = −0.530, *p* = 1.3 × 10^-3^). Among older subjects, PVALB mRNA was downregulated in PVALB cells in the Batiuk cohort (log_2_FC = −0.627, *p* = .015). SST mRNA was significantly downregulated in the McLean (log_2_FC = −1.854, *p* = .009) and Batiuk (log_2_FC = −1.485, *p* = 2.93 × 10^-3^) datasets but upregulated in the Fröhlich dataset (log_2_FC = 2.008, *p* = .0091). VIP mRNA was significantly upregulated in the MSSM (log_2_FC = 0.732, *p* = .022) and Fröhlich (log_2_FC = 0.734, *p* = .0075) datasets, whereas the Batiuk dataset showed significant downregulation (log_2_FC = −1.036, *p* = .0011). Thus, no single dataset recapitulated the full meta-analytic pattern, and even within a cohort, the direction of change was sometimes inverted between age strata.

When we expanded this DE meta-analysis to additional marker genes (Figure S13), we found no transcriptional changes indicative of broad up- or downregulation of multiple cell type–specific marker genes. The sole exception was VIP cells in the <70-year stratum, where 5 of 10 marker genes were significantly downregulated, hinting at a possible erosion of VIP cell transcriptional identity among younger individuals with SCZ.

Taken together, our DE meta-analysis points to selective, age-restricted transcriptional repression of SST and VIP neurons in younger individuals with SCZ. In contrast, PVALB mRNA transcriptional changes remained inconsistent between cohorts. These findings highlight the need for larger, harmonized datasets to more precisely resolve cell-intrinsic changes among GABAergic neurons in SCZ.

## Discussion

**Figure 7:**
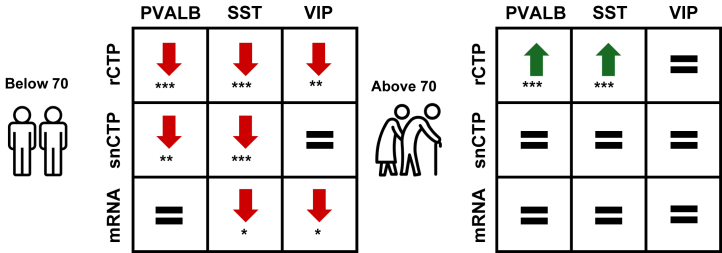
Age-dependent alterations in cortical GABAergic neuron subtypes in SCZ. Summary schematic of SCZ-associated changes in PVALB-, SST-, and VIP-expressing neurons across 3 measures: rCTP from bulk RNA sequencing deconvolution, snCTPs, and cell-intrinsic marker mRNA expression for eponymous marker genes (mRNA). Data are stratified by donor age at death: below 70 years (left) and 70 years and above (right). Red downward arrows indicate significant decreases in SCZ cases versus controls, green upward arrows indicate significant increases, and = denotes no significant difference. Asterisks denote false discovery rate–adjusted *p* values: ∗*p*_FDR_ < .10, ∗∗*p*_FDR_ < .05, ∗∗∗*p*_FDR_ < .01. In younger donors, PVALB and SST cells show reduced proportions (rCTP and snCTP), and SST and VIP cells show reduced mRNA expression; VIP proportions (snCTP) remain unchanged. In older donors, PVALB and SST proportions (rCTP) are significantly increased, with no significant changes in snCTP or mRNA for any subtype. GABAergic, gamma-aminobutyric acidergic; mRNA, messenger RNA; PVALB, parvalbumin; rCTP, relative cell-type proportion; SCZ, schizophrenia; snCTP, single-nucleus CTP; SST, somatostatin; VIP, vasoactive intestinal peptide.

Leveraging 14 bulk- and cell type–resolved RNA-seq datasets from 8 institutional cohorts, we showed that SCZ is characterized by pronounced, age-dependent alterations in key GABAergic cell types. In individuals who were <70 years at death, PVALB and SST rCTPs were markedly reduced in SCZ cases relative to controls. In contrast, among individuals aged ≥70 years at death, these same rCTPs were not significantly changed in individuals with SCZ or were modestly increased relative to control individuals in some cohorts. snRNA-seq and FISH stereology replicated these age-dependent trends, namely demonstrating robust reductions in proportions of PVALB and SST cells among younger individuals with SCZ but nonsignificant differences among older individuals. Complementary DE meta-analyses using snRNA-seq and LCM-seq data revealed selective downregulation of SST and VIP marker mRNAs in younger SCZ cases, whereas PVALB mRNA shows no change in SCZ that is robust across cohorts. Figure 7 synthesizes these findings across modalities and age strata.

What might explain the age dependence of reductions in PVALB and SST cell proportions in SCZ? First, as reported previously by us and others (22,23), healthy aging is accompanied by a cross-sectional reduction in GABAergic cell proportions. Second, our analyses indicate that SCZ cases begin adulthood with a lower baseline proportion of PVALB and SST neurons relative to age-matched controls. However, the age-associated cross-sectional decline in these cells among SCZ cases tended to be much more shallow than in controls. Thus, the combination of a lower starting point and flatter slope in SCZ means that the case-control gap was widest in early adulthood, exactly when postmortem studies of early and midlife SCZ report interneuron loss (26, 27, 28). However, this difference in PVALB and SST proportions between SCZ cases and controls progressively narrows with advancing age, even reversing in some cohorts, after about 70 years of age at death. Thus, the modest late-life increase that we detected in bulk rCTPs is best interpreted as relatively sparing; controls continue to lose PVALB and SST cells, while the loss among SCZ cases plateaus. These findings are consistent with an interpretation of SCZ as resembling an accelerated or anticipated aging phenotype (36,60).

Among our most perplexing findings is the observed increase or preservation in GABAergic neuron proportions in older donors with SCZ in certain cohorts, such as the MSSM. This does not suggest that these neurons are proliferating or growing in the brains of these individuals; instead, this likely represents a relative preservation of these cells compared with the typical loss of these cells observed in healthy aging (22,23). In other words, older cases with SCZ exhibit less loss of these neurons than expected compared with age-matched healthy controls, who would normally exhibit a more pronounced age-related decline in these cells. One explanation for this finding could be potential neuroprotective effects associated with long-term antipsychotic medication use in individuals with SCZ (61,62). Alternatively, this finding may reflect a form of survivorship bias, where those donors with SCZ who live into their 70s and beyond may represent an especially healthy subset of the broader SCZ population and thus may be less affected by typical neurodegenerative processes.

Our most clinically impactful finding is that donors with an earlier age of SCZ onset displayed fewer PVALB and SST cells relative to donors with a later age of SCZ onset, suggesting that the developmental stage at which SCZ emerges considerably shapes the degree of neuron cellular pathology. Early-onset SCZ is strongly linked to more severe clinical outcomes, including greater cognitive impairment, more debilitating negative symptoms, and increased hospitalization (7, 8, 9, 10). While our analyses cannot disentangle the causal chain of events, it is intriguing to hypothesize that vulnerability of PVALB and SST neurons may in fact contribute in part to these symptoms. This would directly implicate interneuron vulnerability in poorer health outcomes and suggest that interventions aimed at preserving PVALB/SST integrity may be especially beneficial for younger or more severely affected individuals.

By leveraging snRNA-seq and LCM-seq resources, we conducted an age-stratified, cell type–specific DE meta-analysis that both confirms and refines earlier molecular observations of reduced PVALB and SST mRNA in SCZ (6,11,28, 29, 30, 31). Among donors <70 years, SST and VIP neurons showed robust downregulation of their eponymous marker transcripts, with minimal between-study heterogeneity. In contrast, our meta-analyses suggest that PVALB mRNA was not significantly reduced in PVALB cells, in part because cohort estimates were highly heterogeneous (*I*^2^ ≈ 70%); while the McLean and Pitt datasets displayed the expected PVALB mRNA downregulation, other datasets did not. These results echo previous reports of cortical interneuron dysfunction (6,11,28, 29, 30, 31) but pinpoint SST and VIP transcriptional changes as the most reproducible signal once cohort heterogeneity is accounted for. Importantly, the coordinated reductions in rCTPs seen in bulk deconvolution and snRNA-seq, together with SST/VIP transcript loss, support a dual-mechanism model for early-life SCZ: vulnerable PVALB/SST cells are depleted, and the remaining SST/VIP population undergoes additional transcriptional remodeling. In donors ≥70 years, DE effects become heterogeneous and largely nonsignificant, mirroring the flattened proportion differences in late life.

Despite the strengths of our study, several limitations warrant acknowledgment. First, our cell-type taxonomy, derived from snRNA-seq data of healthy donors (44,50), may not fully represent the cellular landscape of SCZ or account for age-related changes in disease states. Second, bulk RNA-seq deconvolution provides a useful but indirect proxy for cell proportions (63), despite our careful benchmarking against gold-standard snRNA-seq cell counts and stereology-derived cell densities. Third, our study aggregates effects across heterogeneous cohorts with a number of differences, including in genetic ancestry, sex balance, cortical region sampled, antipsychotic medication status, diagnostic criteria, and technical factors such as RNA quality, PMI, and sequencing chemistry, can all influence cell proportions and DE estimates. For example, the older MSSM cohort contributes most of the late-life preservation signal, whereas datasets collected from younger cohorts (e.g., GVEX, Pitt, NIMH_HBCC) demonstrate the greatest earlier adulthood GABAergic cell deficits. Fourth, while our study illuminates the age-dependent effects of SCZ on GABAergic neurons, it does not fully explore the potential roles of other cell types, such as pyramidal cells or astrocytes, which are also critical to SCZ pathology (27,64). Similarly, our study does not consider further subdividing PVALB or SST neurons, and we note that recent studies have identified upper-layer PVALB cells as being specifically vulnerable in SCZ (28). Fifth, our reliance on postmortem data limits our ability to infer dynamic pathological processes occurring within individuals over their lifetimes. Finally, diagnosis- and age-related factors that affect tissue quality, most notably perimortem hypoxia and the consequent reduction in brain pH (58,59), could independently affect interneuron marker expression, thereby influencing the proportional shifts that we observe.

Our study has several important implications for future research. The age-dependent changes in neuron proportions suggest that interventions may need to be tailored to different life stages in SCZ. Additionally, the potential neural resilience that we observed in certain cohorts comprising older SCZ donors warrants further investigation; identifying potential factors that contribute to this resilience, if confirmed, could lead to new therapeutic strategies aimed at mitigating the severity of SCZ. The observed variability across cohorts highlights the need for ongoing large-scale, demographically diverse consortia, such as PsychENCODE (42,65), that can account for demographic and clinical differences. Furthermore, given the potential impact of neuron changes on cortical microcircuit function, future studies using computational models (66,67) incorporating our cell type–specific effect sizes, could investigate how cellular alterations in these specific cell types affect information processing and cognitive functions in patients with SCZ. Understanding these mechanisms better could provide deeper insights into the cognitive symptoms of SCZ and inform the development of interventions aimed at restoring normal cortical function. Beyond SCZ, dysregulation of PVALB-, SST-, and VIP-expressing interneurons has been implicated in a range of neuropsychiatric and neurodegenerative conditions including bipolar disorder, major depression, autism spectrum disorder, and Alzheimer’s disease (50,68, 69, 70). Situating our SCZ-focused findings within this broader context highlights both the transdiagnostic relevance of GABAergic imbalance and the potential for shared therapeutic targets that cut across traditional diagnostic boundaries.

In conclusion, our work offers quantitatively grounded evidence for age-dependent dysregulation of cortical GABAergic neurons in SCZ and highlights the need for multifaceted, age-aware approaches in future mechanistic and translational work aimed at understanding and treating this disorder.

## Supplementary Material

Supplemental Data

Key Resources Table
