# Supplementary material for: Cortical GABAergic Neuron Dysregulation in Schizophrenia Is Age Dependent
Source: Biol Psychiatry Glob Open Sci. 2025 Sep 8;6(1):100606. doi: 10.1016/j.bpsgos.2025.100606 (PMC12556227; doi:10.1016/j.bpsgos.2025.100606)
Supplement: Figures S1–S13 and Tables S1–S5 [file mmc1.pdf]

SUPPLEMENTARY INFORMATION

Cortical GABAergic Neuron Dysregulation in Schizophrenia Is Age Dependent

Kiss *et al.*

Supplementary Figures

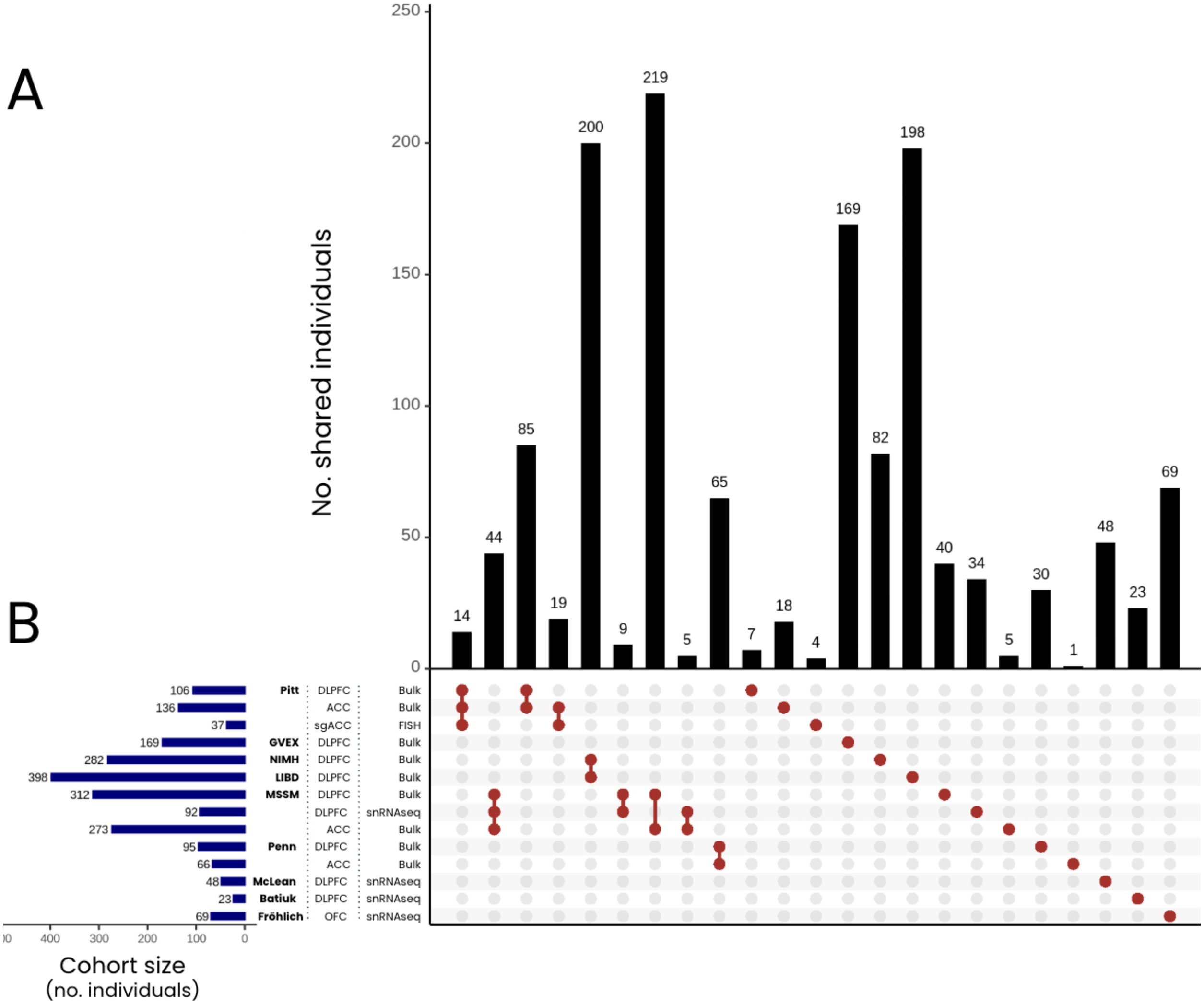

Figure S1. Cohort overlap across schizophrenia transcriptomic datasets.
UpSet plot showing the overall cohort / brain bank, dataset size, and subject overlap for each dataset included in the analysis, grouped by brain region and technology (bulk RNA-seq, snRNA-seq, or FISH / LCMseq).

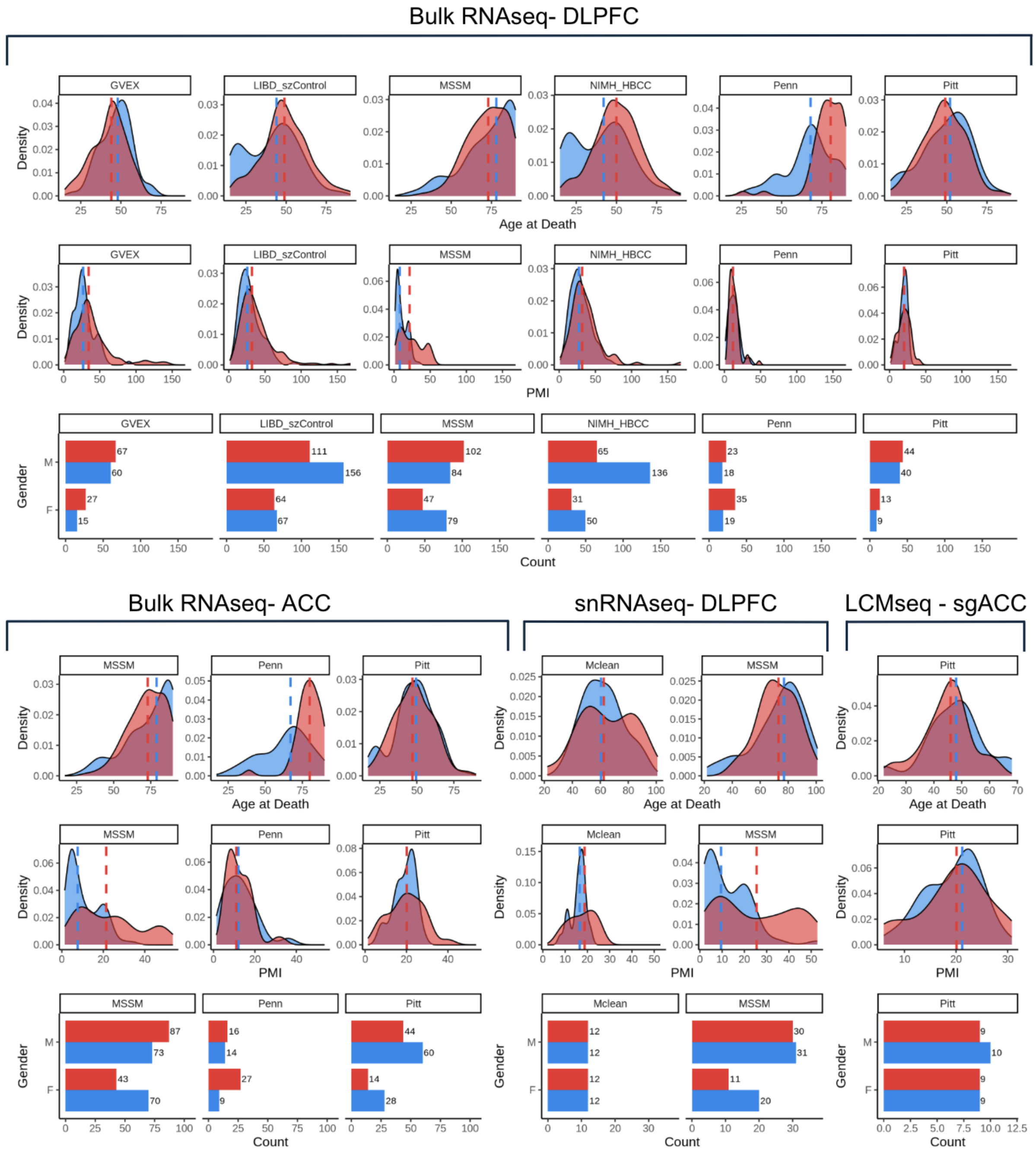

Figure S2. Cohort metadata distributions across transcriptomic datasets. *Density plots of age at death (top) and postmortem interval (PMI; center) are shown for each PsychEncode based dataset, with dashed vertical lines indicating median values listed in Table 1. Horizontal bar plots (bottom) display the distribution of gender, with the number of individuals for each gender indicated at the end of each bar. Data are derived from multiple cohorts spanning bulk RNA-seq (DLPFC and ACC), single-nucleus RNA-seq, and FISH + LCMseq.*

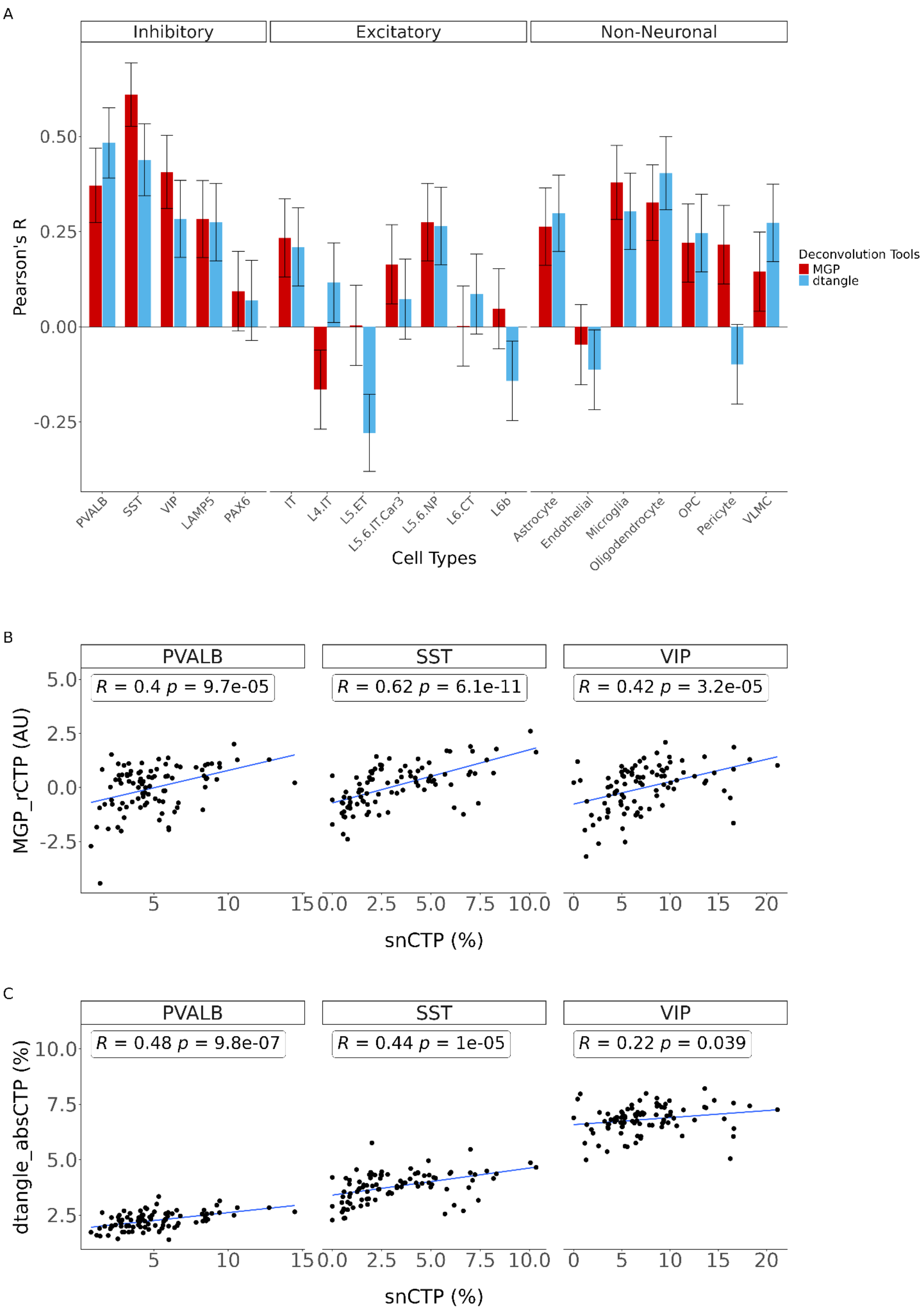

Figure S3. Assessment of cell type proportion predictions from MGP and dtangle against snCTPs from matched donors. *(A) Barplot depicting Pearson's correlations (R) between MGP and dtangle-derived bulk deconvolution estimates and single-nucleus cell type proportions. Error bars represent the standard error for each Pearson correlation. (B) Scatter plots illustrating*

the correlation between snCTPs (x-axis, %) and MGP-derived relative cell type proportions (rCTPs; y-axis, arbitrary units, AU). Here, the MGP algorithm was used but when explicitly removing the eponymous marker gene for each cell type (e.g., PVALB mRNA for PVALB cells). Inset values indicate Pearson's correlation coefficient (R) and the corresponding p-value. (C) Scatter plots illustrate the correlation between snCTPs (x-axis, %) and dtangle-derived absolute cell type proportions (absCTPs; y-axis, %). The dtangle algorithm used a marker gene list that excluded the eponymous gene for each cell type (as in B).

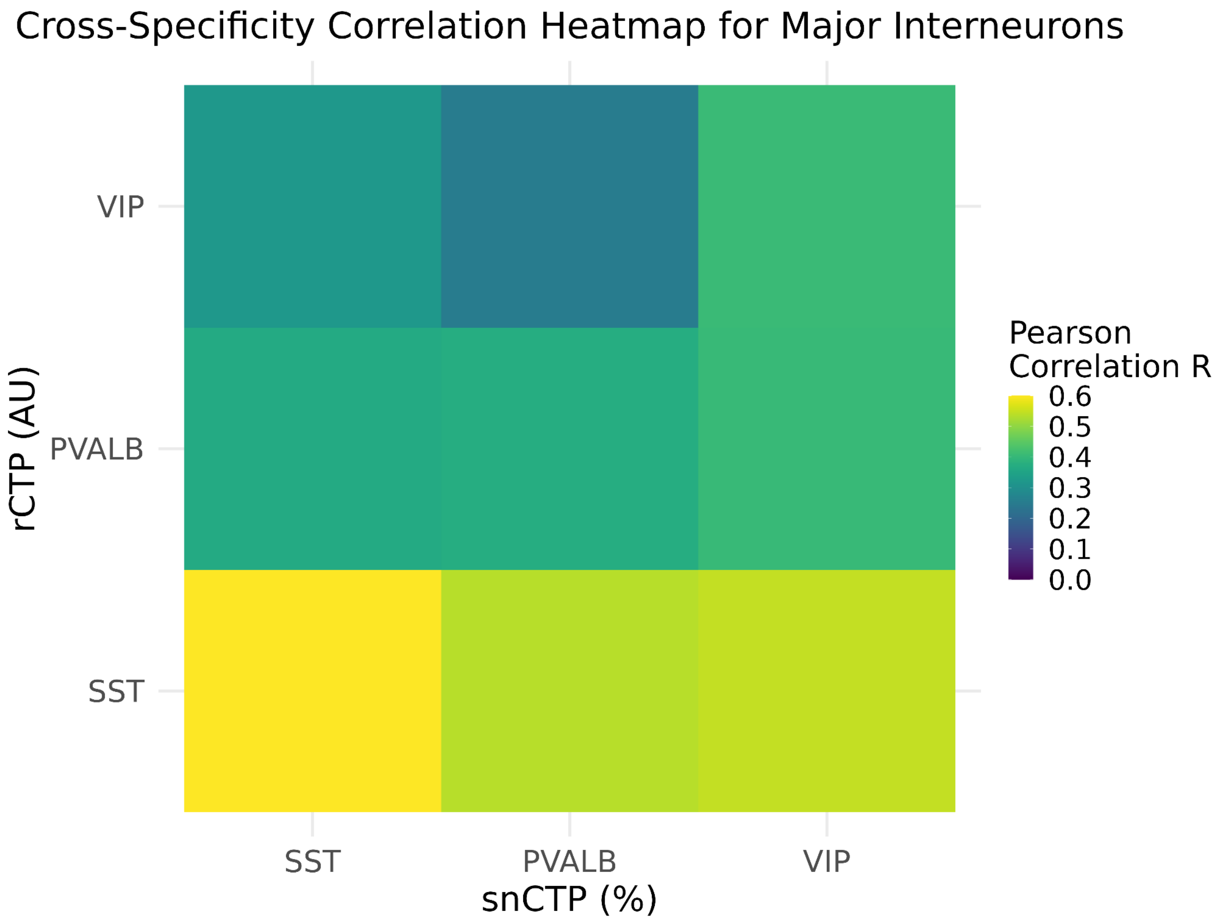

Figure S4. Cross-specificity analysis for major GABAergic cells. The heatmap shows Pearson Correlation Coefficients (*R*) comparing bulk-derived relative cell type proportion (rCTP) estimated via MGP on the y-axis with single-nucleus cell type proportion (snCTPs) on the x-axis between each pair of cell types.

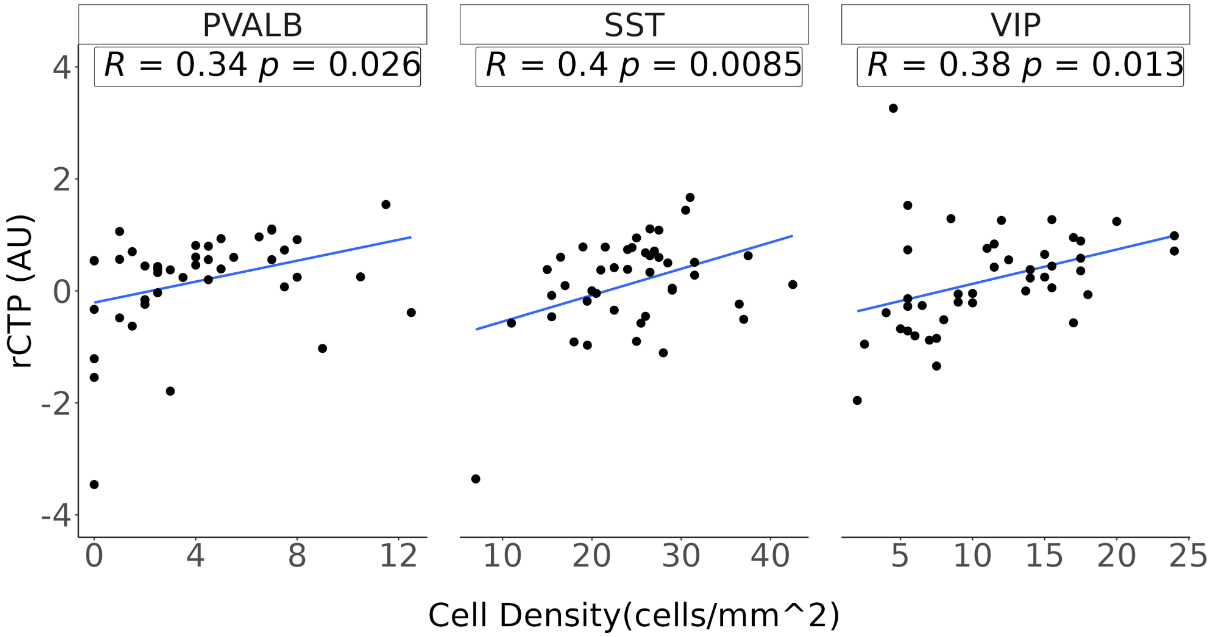

Figure S5. Correlation of MGP-derived rCTPs with microscopy-based cell densities for three major neuron types in ACC from matched donors. *Scatterplots showing the correlation between fluorescence in situ hybridization-based cell densities (expressed as number of labelled cells per millimeter squared, x-axis) and relative cell type proportions (rCTPs; y-axis, arbitrary units, AU) derived from bulk tissue deconvolution via the MGP algorithm. Inset values show Pearson's correlation coefficient (R) and corresponding p-values.*

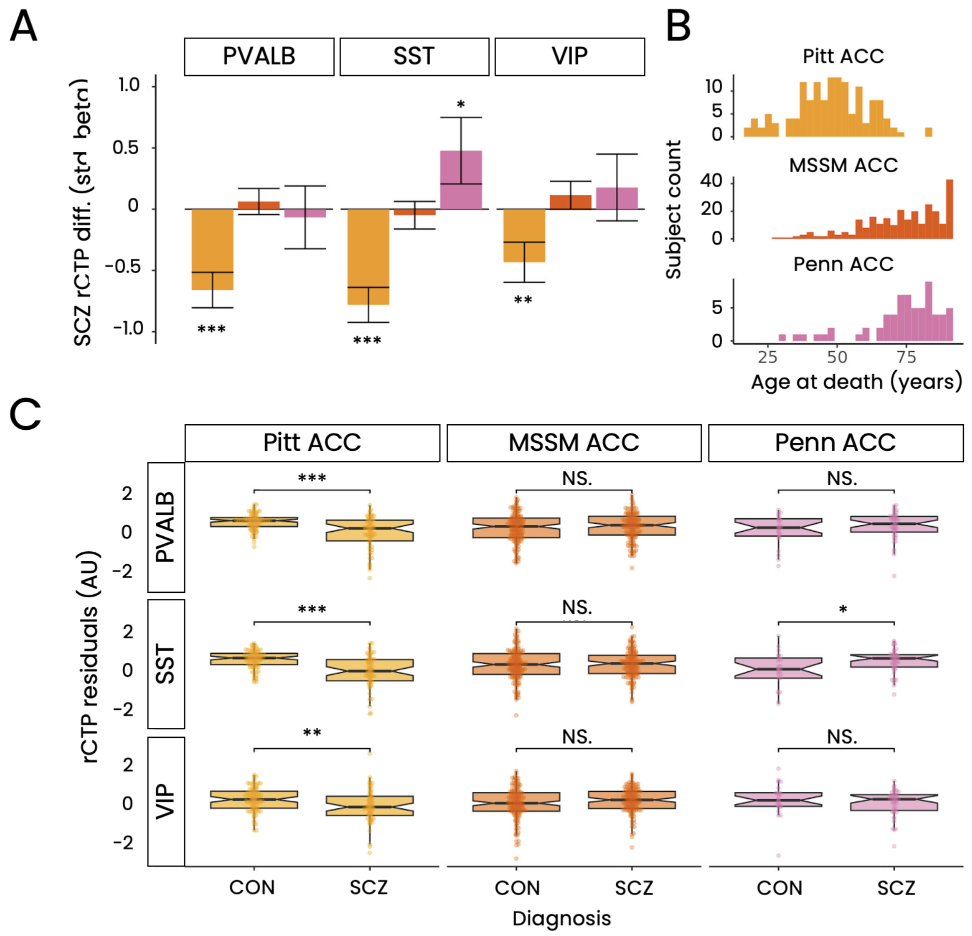

Figure S6. ***Differences in ACC GABAergic neuron proportions between schizophrenia and controls.*** *(A) Bar plots showing standardized beta coefficients (β) for differences in relative cell type proportions (rCTPs) of PVALB, SST, and VIP neurons between schizophrenia (SCZ) and control (C) groups across three bulk ACC RNA-seq datasets. Positive values indicate increased proportions in SCZ, while negative values indicate decreases. Error bars represent standard error, and asterisks indicate significance based on false discovery rate FDR (* = 0.1, ** = 0.05, *** = 0.01). (B) Age at death distribution for each dataset. Datasets are ordered by increasing mean age in years. (C) Box plots of residualized rCTPs, after controlling for demographic and technical covariates, for PVALB, SST, and VIP neurons across datasets. Asterisks reflect significance from panel (A), with "NS" for non-significant comparisons.*

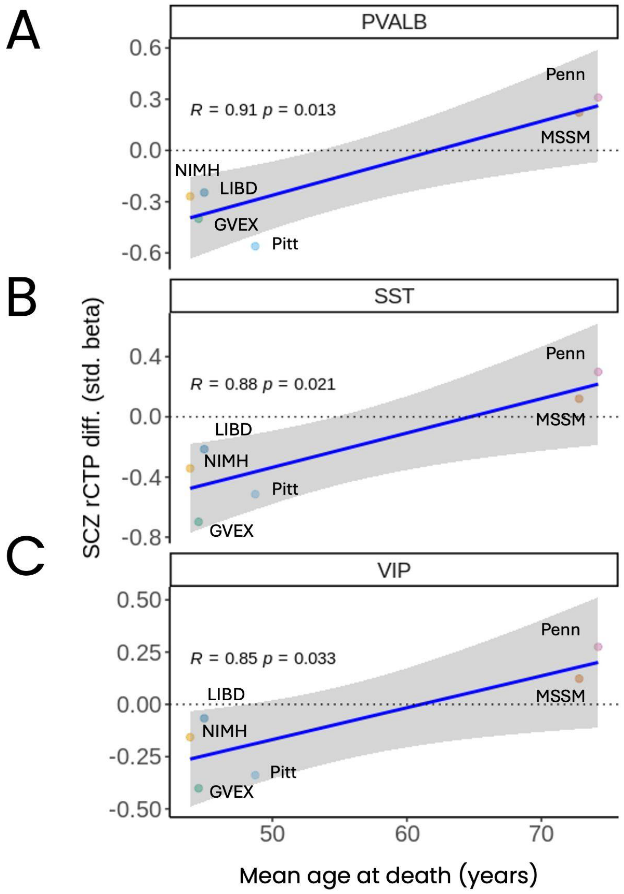

Figure S7. Correlating average DLPFC dataset age at death with SCZ-rCTP association strength. (A-C) Associations between the degree to which SCZ is associated with reduced or increased cell type proportions of PVALB, SST, and VIP cells and mean dataset age at death in size PsychEncode DLPFC datasets. Y axis shows standard beta coefficient values (as in Figure 2A) based on linear modelling and accounting for covariates. X-axis shows the mean age at death for each cohort. Cohorts are marked and labelled on each plot. Inset line represents linear model fit, and shaded area indicates standard error.

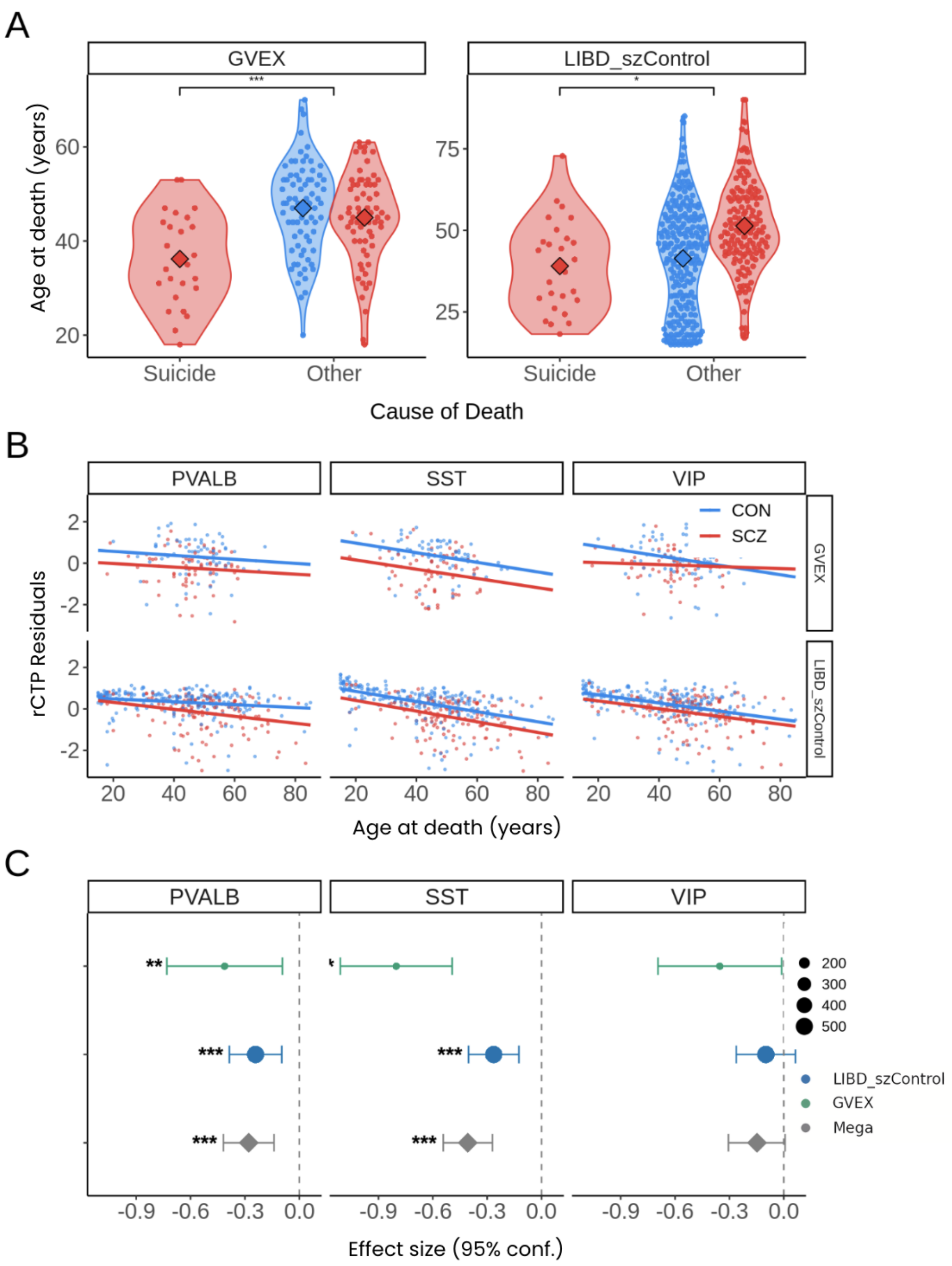

Figure S8. Associations between cause of death and age at death in schizophrenia and control samples. (A) Violin plots show the distribution of age at death in individuals who died by suicide or other causes, stratified by primary diagnosis (schizophrenia vs. control) in the GVEX and LIBD_szControl datasets. Individual dots represent per-subject data points and diamonds indicate group means. Statistical comparisons between suicide and other deaths were performed using the Wilcoxon rank-sum test, with significance levels denoted by asterisks. (B) Scatterplots illustrate the association between age at death and residualised proportions of PVALB, SST, and VIP neurons in schizophrenia (SCZ) versus controls after excluding subjects with suicide annotated as a cause of death. (C) Forest plots of the schizophrenia effect (β) on rCTPs after excluding subjects with suicide annotated as a cause of death. Points give the regression coefficient from cohort-specific linear models (covariates: age, sex, PMI, RIN);

Diamonds represent the pooled (mega-analysis) estimate; circles represent cohort-specific estimates. Horizontal bars are 95 % confidence intervals, and point size is proportional to cohort sample size. Asterisks positioned to the left (negative effect) or right (positive effect) of each bar follow the same FDR thresholds as in panel A.

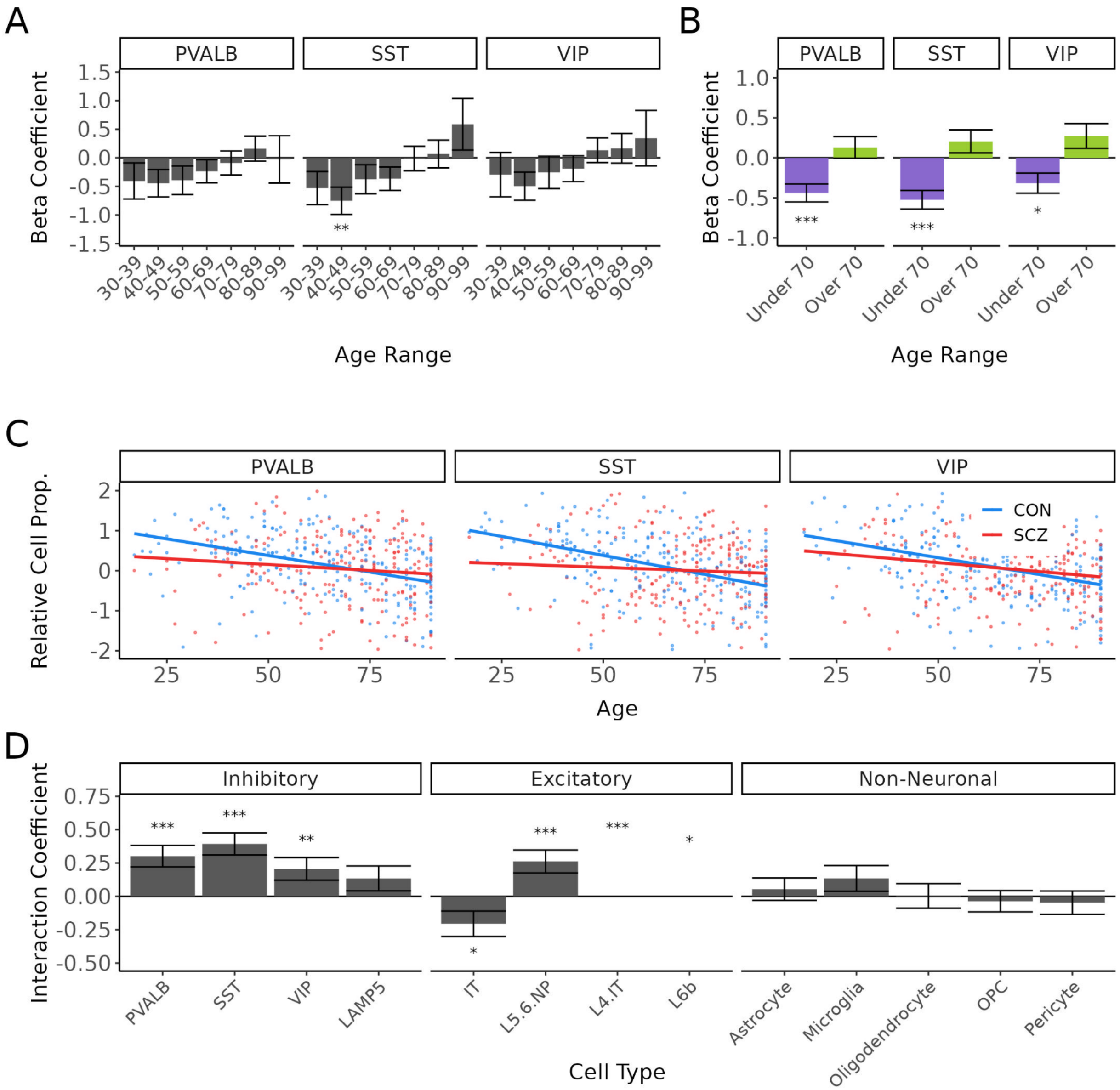

Figure S9. The effect of age at death on schizophrenia-associated differences in ACC neuron proportions. *(A) Scatterplots showing the relationship between age at death and residualized proportions of PVALB, SST, and VIP neurons in schizophrenia (SCZ) and controls. Each point represents an individual bulk RNA-seq sample, aggregated across ACC datasets, with lines indicating the best linear fit for each group. (B) Bar plots displaying the effect of SCZ*

on neuron proportions, binned by decade of age at death. The y-axis shows standardized beta coefficients from linear models adjusting for covariates. Error bars represent standard error, and asterisks denote significance based on FDR thresholds (= 0.1, ** = 0.05, *** = 0.01). (C) Bar plots showing SCZ effects on neuron proportions for donors above and below 70 years. The y-axis shows beta coefficients from linear models, with error bars and FDR-based significance as in panel (B). (D) Bar plots of the interaction between age at death and diagnosis on cell type proportions. Interaction coefficients indicate how the effect of SCZ on cell type proportions changes with age; positive coefficients show that SCZ-associated differences in cell type proportions increase with age. Error bars represent standard error, and asterisks denote significance based on FDR thresholds.*

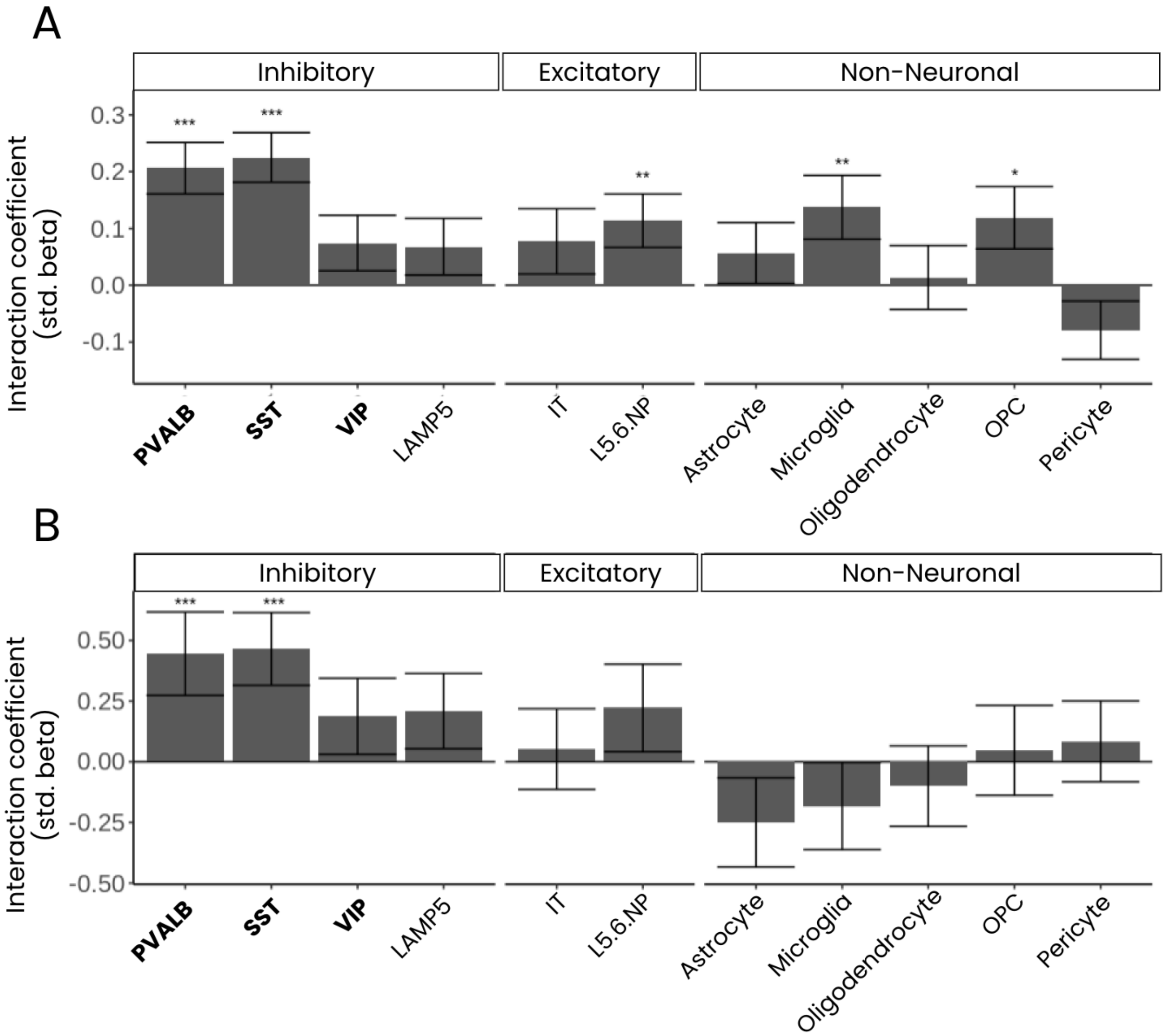

Supplementary Figure S10: Age × Diagnosis interaction effects on cell proportions across neocortical cell types. Panels show standardized interaction coefficients (β) for the age × schizophrenia term from linear models predicting cell-type proportions in (A) bulk RNA-seq deconvolved relative cell-type proportions (rCTPs) and (B) single-nucleus cell-type proportions (snCTPs). Cell types are grouped by class: Inhibitory (PVALB, SST, VIP, LAMP5), Excitatory (IT, L5/6.NP) and Non-Neuronal (Astrocyte, Microglia, Oligodendrocyte, OPC, Pericyte). Bars represent the pooled (mega-analysis) interaction estimate across all cohorts; error bars indicate ± standard error. Positive interaction coefficients signify that the schizophrenia–control difference in that cell-type proportion grows with increasing age at death, whereas negative coefficients indicate the difference diminishes with age. Asterisks denote FDR-adjusted significance (* ≤ 0.10; ** ≤ 0.05; *** ≤ 0.01).

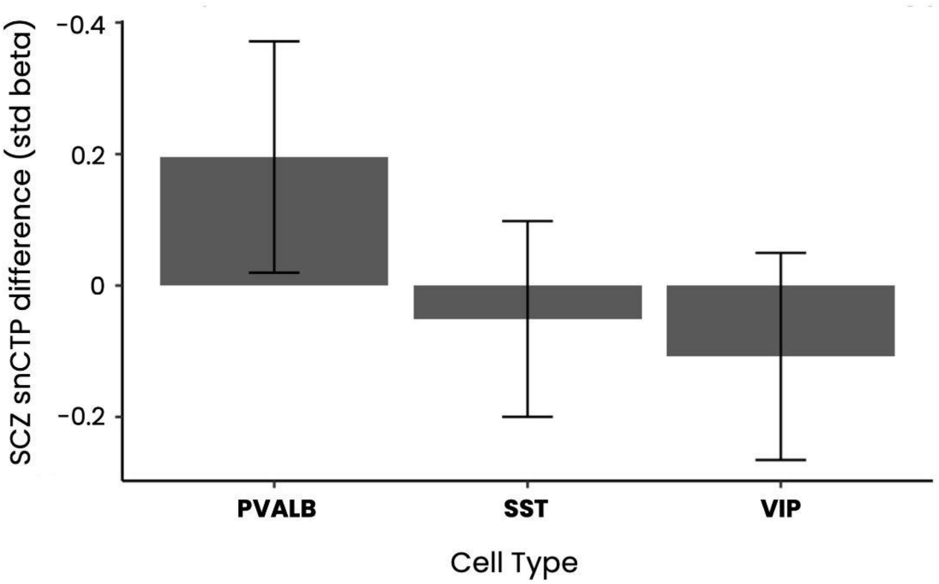

Figure S11. Schizophrenia-associated differences in single-nucleus cell type proportions of DLPFC interneuron subtypes without stratified age groups. *Bar plots of SCZ effects on snCTPs without stratifying for age. The y-axis shows standardized beta coefficients from linear models adjusting for covariates. Error bars represent standard error, and asterisks indicate significance based on FDR thresholds, with no asterisk indicating FDR > 0.01.*

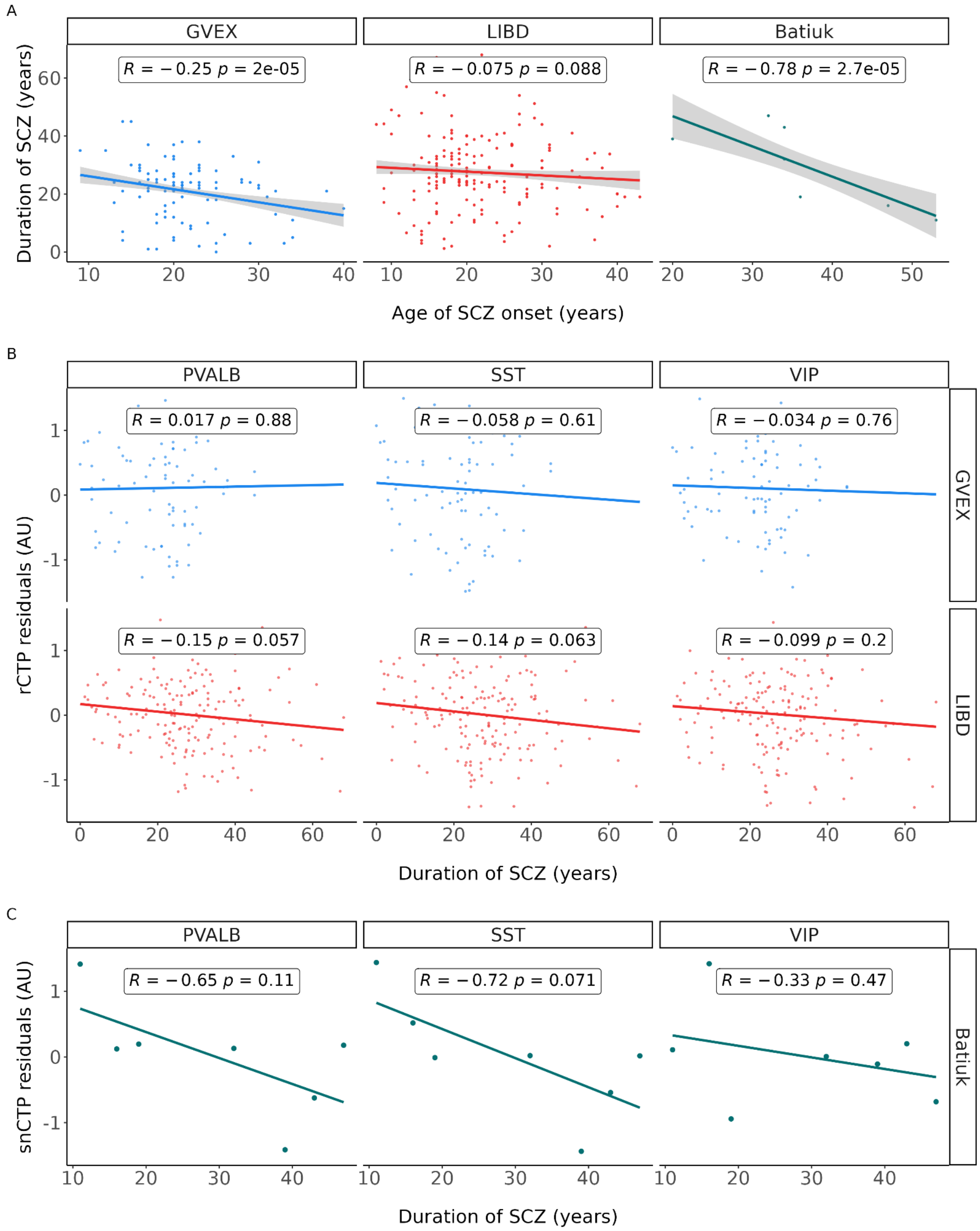

Figure S12. Associations between duration and age of schizophrenia onset with GABAergic neuron cell type proportions. *(A) Relationships between SCZ duration of illness and age of SCZ onset across three datasets (GVEX, LIBD, and Batiuk) where onset information was available. Each dot represents a single donor; the Y-axis shows SCZ duration of illness, and the X-axis shows age of SCZ onset. Lines indicate best linear fits with shaded 95% confidence intervals. Pearson's correlation coefficients (R) and p-values are shown in each facet. (B) Associations between SCZ duration of illness and residualized relative cell type proportions (rCTPs) of PVALB, SST, and VIP neurons in GVEX and LIBD bulk RNA-seq datasets. rCTPs were adjusted for age at death, RIN, PMI, and other covariates. Each dot represents an individual sample, colored by dataset, with linear trend lines and associated R*

and p-values shown. (C) Associations between SCZ duration of illness and snCTPs for the same neuron subtypes in the Batiuk dataset. snCTPs were adjusted for age at death, PMI, and other covariates Each dot corresponds to a donor, and inset correlations represent Pearson's R and p-values.

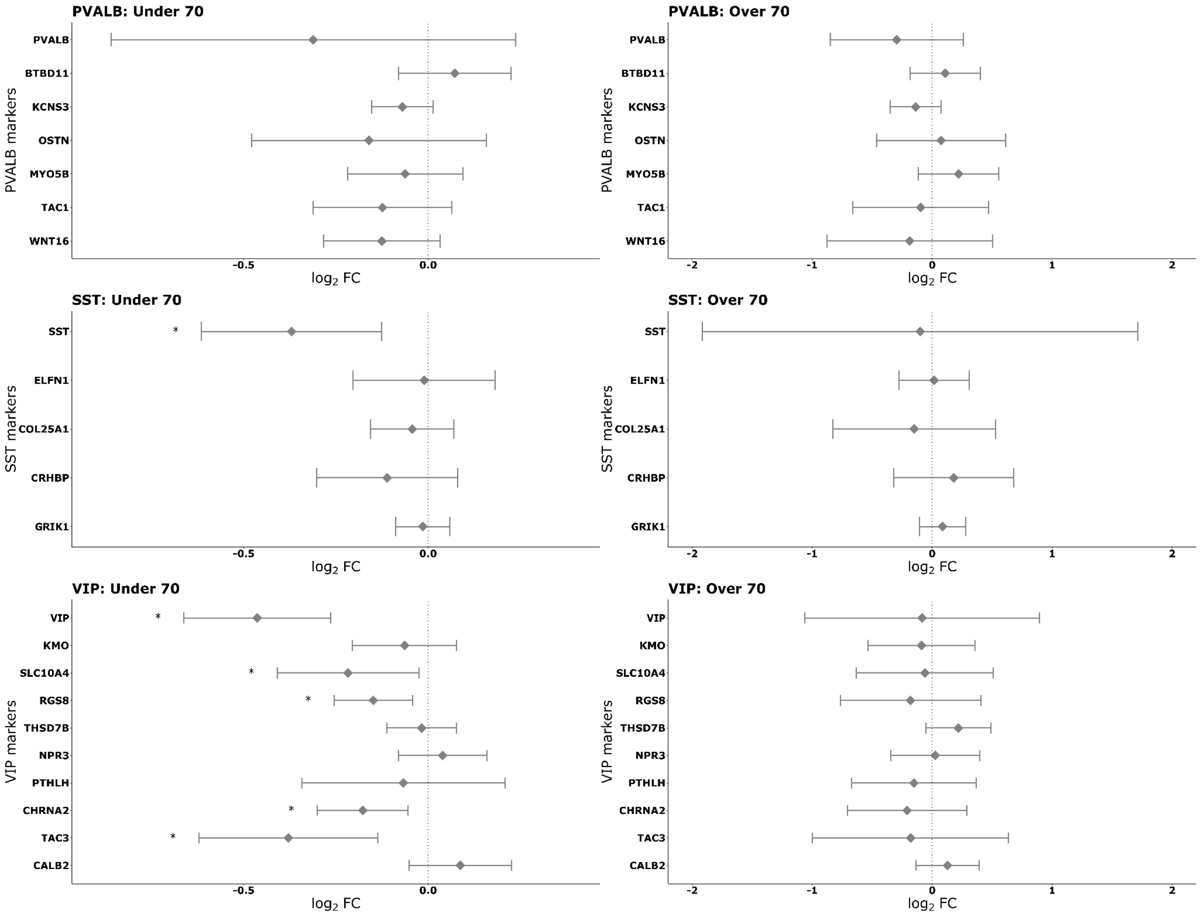

Supplementary Figure S13. Differential expression of neuron subtype marker genes in schizophrenia: Meta-analyzed results across age groups. Forest plots display meta-analyzed $\log_2$ fold changes in SCZ vs. control for marker genes of PVALB (A), SST (B), and VIP (C) neurons, derived from differential expression meta-analysis. Results are stratified by age at death (Under 70 vs. Over 70 years). Only the meta-analysis estimates are shown, combining data across five independent cell type-specific RNAseq datasets (McLean, MSSM, Batiuk, Fröhlich, Pitt) using weighted effect size modeling. Forest plots display $\log_2$ fold changes with 95% confidence intervals for each cell type specific marker gene, visualized by cell type and age group. Asterisks indicate nominal significance ($p < 0.05$).

Supplementary Tables

Table S1. *Marker genes table for three major GABAergic neurons.*

Cell Types	Marker Genes									
PVALB	PVALB	WNT16	TAC1	MYO5B	OSTN	KCNS3	BTBD11			
SST	SST	GRIK1	CRHBP	COL25A1	CORT	ELFN1				
VIP	VIP	CALB2	TAC3	CHRNA2	PTHLH	NPR3	THSD7B	RGS8	SLC10A4	KMO

Table S2. Summary of SCZ-related changes by age class in PVALB

Cohort	Brain Area	Assay	Cell Type	Cases	Controls	Age Group	p.value	padj	Effect
MSSM	DLPFC	Bulk	PVALB	149	163	Below 70	3.68E-01	6.54E-01	NA
Penn	DLPFC	Bulk	PVALB	58	37	Below 70	4.75E-01	7.36E-01	NA
Pitt	DLPFC	Bulk	PVALB	57	49	Below 70	4.38E-03	2.83E-02	DOWN
NIMH	DLPFC	Bulk	PVALB	96	186	Below 70	2.01E-03	1.52E-02	DOWN
LIBD	DLPFC	Bulk	PVALB	175	223	Below 70	4.57E-04	4.18E-03	DOWN
GVEX	DLPFC	Bulk	PVALB	94	75	Below 70	1.35E-02	6.91E-02	DOWN
MSSM_ACC	ACC	Bulk	PVALB	130	143	Below 70	3.59E-01	5.76E-01	NA
Penn_ACC	ACC	Bulk	PVALB	43	23	Below 70	3.51E-01	5.70E-01	NA
Pitt_ACC	ACC	Bulk	PVALB	58	88	Below 70	3.52E-05	6.26E-04	DOWN
MSSM (snRNA)	DLPFC	Single Nucleus	PVALB	18	17	Below 70	1.58E-02	3.23E-01	DOWN (trending)
McLean	DLPFC	Single Nucleus	PVALB	14	18	Below 70	7.09E-01	9.92E-01	NA
Batiuk	DLPFC	Single Nucleus	PVALB	5	10	Below 70	1.18E-01	9.43E-01	NA
Fröhlich	OFC	Single Nucleus	PVALB	32	29	Below 70	7.07E-01	8.68E-01	NA
Mega (snRNA)		Single Nucleus	PVALB	69	74	Below 70	8.38E-03	2.38E-02	DOWN
MSSM	DLPFC	Bulk	PVALB	149	163	Above 70	4.34E-05	5.39E-04	UP
Penn	DLPFC	Bulk	PVALB	58	37	Above 70	6.82E-02	2.46E-01	UP (trending)
Pitt	DLPFC	Bulk	PVALB	57	49	Above 70	NA	NA	NA
NIMH	DLPFC	Bulk	PVALB	96	186	Above 70	1.89E-01	4.56E-01	NA
LIBD	DLPFC	Bulk	PVALB	175	223	Above 70	2.72E-02	1.22E-01	DOWN (trending)
MSSM_ACC	ACC	Bulk	PVALB	130	143	Above 70	2.41E-01	4.76E-01	NA
Penn_ACC	ACC	Bulk	PVALB	43	23	Above 70	5.85E-01	7.85E-01	NA
Pitt_ACC	ACC	Bulk	PVALB	58	88	Above 70	NA	NA	NA
MSSM (snRNA)	DLPFC	Single Nucleus	PVALB	23	34	Above 70	1.27E-03	5.02E-02	UP
McLean	DLPFC	Single Nucleus	PVALB	10	6	Above 70	6.68E-01	9.92E-01	NA
Batiuk	DLPFC	Single Nucleus	PVALB	4	4	Above 70	6.08E-01	9.31E-01	NA
Fröhlich	OFC	Single Nucleus	PVALB	4	4	Above 70	8.62E-01	9.67E-01	NA
Mega (snRNA)		Single Nucleus	PVALB	41	48	Above 70	1.22E-01	2.30E-01	NA

Table S3. Summary of SCZ-related changes by age class in SST

Cohort	Brain Area	Assay	Cell Type	Cases	Controls	Age Group	p.value	padj	Effect
MSSM	DLPFC	Bulk	SST	149	163	Below 70	3.72E-01	6.57E-01	NA
Penn	DLPFC	Bulk	SST	58	37	Below 70	3.36E-01	6.36E-01	NA
Pitt	DLPFC	Bulk	SST	57	49	Below 70	1.31E-02	6.74E-02	DOWN
NIMH	DLPFC	Bulk	SST	96	186	Below 70	1.76E-07	3.16E-06	DOWN
LIBD	DLPFC	Bulk	SST	175	223	Below 70	6.22E-05	7.29E-04	DOWN
GVEX	DLPFC	Bulk	SST	94	75	Below 70	3.98E-04	3.82E-03	DOWN
MSSM_ACC	ACC	Bulk	SST	130	143	Below 70	1.44E-01	3.62E-01	NA
Penn_ACC	ACC	Bulk	SST	43	23	Below 70	9.01E-01	9.61E-01	NA
Pitt_ACC	ACC	Bulk	SST	58	88	Below 70	1.74E-06	4.85E-05	DOWN
MSSM (snRNA)	DLPFC	Single Nucleus	SST	18	17	Below 70	1.94E-03	1.12E-01	DOWN (trending)
McLean	DLPFC	Single Nucleus	SST	14	18	Below 70	3.15E-01	9.78E-01	NA
Batiuk	DLPFC	Single Nucleus	SST	5	10	Below 70	2.41E-01	9.43E-01	NA
Fröhlich	OFC	Single Nucleus	SST	32	29	Below 70	1.72E-02	2.47E-01	DOWN (trending)
Mega (snRNA)		Single Nucleus	SST	69	74	Below 70	5.00E-05	2.64E-04	DOWN
MSSM	DLPFC	Bulk	SST	149	163	Above 70	3.90E-03	2.61E-02	UP
Penn	DLPFC	Bulk	SST	58	37	Above 70	2.59E-01	5.47E-01	NA
Pitt	DLPFC	Bulk	SST	57	49	Above 70	NA	NA	NA
NIMH	DLPFC	Bulk	SST	96	186	Above 70	9.61E-01	9.83E-01	NA
LIBD	DLPFC	Bulk	SST	175	223	Above 70	9.13E-02	2.98E-01	DOWN (trending)
MSSM_ACC	ACC	Bulk	SST	130	143	Above 70	5.27E-01	7.35E-01	NA
Penn_ACC	ACC	Bulk	SST	43	23	Above 70	4.37E-02	1.89E-01	UP (trending)
Pitt_ACC	ACC	Bulk	SST	58	88	Above 70	NA	NA	NA
MSSM (snRNA)	DLPFC	Single Nucleus	SST	23	34	Above 70	1.40E-03	5.02E-02	UP
McLean	DLPFC	Single Nucleus	SST	10	6	Above 70	5.03E-01	9.92E-01	NA
Batiuk	DLPFC	Single Nucleus	SST	4	4	Above 70	1.63E-02	3.92E-01	DOWN (trending)
Fröhlich	OFC	Single Nucleus	SST	4	4	Above 70	5.89E-01	9.32E-01	NA
Mega (snRNA)		Single Nucleus	SST	41	48	Above 70	7.57E-01	8.65E-01	NA

Table S4. Summary of SCZ-related changes by age class in VIP

Cohort	Brain Area	Assay	Cell Type	Cases	Controls	Age Group	p.value	padj	Effect
MSSM	DLPFC	Bulk	VIP	149	163	Below 70	3.38E-01	6.37E-01	NA
Penn	DLPFC	Bulk	VIP	58	37	Below 70	1.84E-01	4.51E-01	NA
Pitt	DLPFC	Bulk	VIP	57	49	Below 70	1.86E-01	4.51E-01	NA
NIMH	DLPFC	Bulk	VIP	96	186	Below 70	3.56E-03	2.40E-02	DOWN
LIBD	DLPFC	Bulk	VIP	175	223	Below 70	1.48E-01	4.12E-01	NA
GVEX	DLPFC	Bulk	VIP	94	75	Below 70	4.96E-02	1.95E-01	DOWN (trending)
MSSM_ACC	ACC	Bulk	VIP	130	143	Below 70	4.81E-01	6.94E-01	NA
Penn_ACC	ACC	Bulk	VIP	43	23	Below 70	6.84E-02	2.46E-01	DOWN (trending)
Pitt_ACC	ACC	Bulk	VIP	58	88	Below 70	1.71E-02	9.82E-02	DOWN
MSSM (snRNA)	DLPFC	Single Nucleus	VIP	18	17	Below 70	1.91E-01	9.47E-01	NA
McLean	DLPFC	Single Nucleus	VIP	14	18	Below 70	1.57E-01	9.40E-01	NA
Batiuk	DLPFC	Single Nucleus	VIP	5	10	Below 70	8.85E-02	9.43E-01	Down (trending)
Fröhlich	OFC	Single Nucleus	VIP	32	29	Below 70	3.96E-01	8.08E-01	NA
Mega (snRNA)		Single Nucleus	VIP	69	74	Below 70	1.23E-01	2.31E-01	NA
MSSM	DLPFC	Bulk	VIP	149	163	Above 70	5.50E-02	2.09E-01	UP (trending)
Penn	DLPFC	Bulk	VIP	58	37	Above 70	2.24E-01	5.07E-01	NA
Pitt	DLPFC	Bulk	VIP	57	49	Above 70	NA	NA	NA
NIMH	DLPFC	Bulk	VIP	96	186	Above 70	2.95E-01	5.88E-01	NA
LIBD	DLPFC	Bulk	VIP	175	223	Above 70	2.25E-01	5.07E-01	NA
MSSM_ACC	ACC	Bulk	VIP	130	143	Above 70	2.98E-01	5.28E-01	NA
Penn_ACC	ACC	Bulk	VIP	43	23	Above 70	1.04E-01	3.03E-01	NA
Pitt_ACC	ACC	Bulk	VIP	58	88	Above 70	NA	NA	NA
MSSM (snRNA)	DLPFC	Single Nucleus	VIP	23	34	Above 70	7.50E-01	9.92E-01	NA
McLean	DLPFC	Single Nucleus	VIP	10	6	Above 70	9.58E-01	1.00E+00	NA
Batiuk	DLPFC	Single Nucleus	VIP	4	4	Above 70	9.31E-01	9.89E-01	NA
Fröhlich	OFC	Single Nucleus	VIP	4	4	Above 70	7.07E-01	9.67E-01	NA
Mega (snRNA)		Single Nucleus	VIP	41	48	Above 70	8.00E-01	8.88E-01	NA

Table S5. Datasets Used for Each Figure in the Manuscript

Dataset / Cohort Name	Brain Area	% Male	Age (SD)	RNA Type	# Case	# Control	Figure 1	Figure 2	Figure 3	Figure 4	Figure 5	Figure 6
MSSM (bulk)	DLPFC	59.6	72.8 (14.6)	Bulk	149	163	✓	✓	✓			
Penn	DLPFC	43.2	74.2 (13.9)	Bulk	58	37		✓	✓			
Pitt	DLPFC	79.2	48.7 (13.9)	Bulk	57	49		✓	✓			
NIMH_HBCC	DLPFC	71.3	43.9 (16.6)	Bulk	96	186		✓	✓			
LIBD_szControl	DLPFC	67.1	44.9 (16.2)	Bulk	175	223		✓	✓		✓	
GVEX	DLPFC	75.1	44.5 (10.3)	Bulk	94	75		✓	✓		✓	
McLean	DLPFC	66.3	63.2 (16.9)	snRNAseq	24	24				✓		✓

MSSM (snRNA)	DLPFC	50.0	72.7 (16.5)	snRNAseq	41	51	✓			✓		✓
Batiuk	DLPFC	52.2	65 (10.3)	snRNAseq	14	9				✓	✓	✓
Fröhlich	OFC	65.2	56 (12.7)	snRNAseq	33	36				✓		✓
Pitt_sgACC FISH+LCMseq	sgACC	50.0	48.3 (9.8)	LCM-seq	18	19				✓		✓
